# Identification of *recG* genetic interactions in *Escherichia coli* by transposon sequencing

**DOI:** 10.1128/jb.00184-23

**Published:** 2023-11-29

**Authors:** Nina J. Bonde, Elizabeth A. Wood, Kevin S. Myers, Michael Place, James L. Keck, Michael M. Cox

**Affiliations:** 1 Department of Biochemistry, University of Wisconsin-Madison, Madison, Wisconsin, USA; 2 Department of Biomolecular Chemistry, University of Wisconsin-Madison, Madison, Wisconsin, USA; 3 Great Lakes Bioenergy Research Center and the Wisconsin Energy Institute, University of Wisconsin-Madison, Madison, Wisconsin, USA; Queen Mary University of London, London, United Kingdom

**Keywords:** RecG, DNA repair, transposon sequencing, *Escherichia coli*

## Abstract

Maintaining the integrity of the genome is of utmost importance for cell division and propagation. In *Escherichia coli*, the RecG protein has been implicated in processing branched recombination intermediates during DNA repair processes, but the primary cellular role(s) of RecG and the repair pathways in which it acts have been difficult to define. To gain additional insight into RecG function, we employed transposon sequencing to identify *recG* genetic interactions and reveal complementary or redundant functions. The strongest hits from the screen were the *dam*, *uvrD*, *rnhA*, *radA*, and *rep* genes. The conditional importance of these five genes in cells lacking *recG* was confirmed using a plasmid-based assay, revealing synthetic lethal interactions for most double deletion strains. Several of the synthetic lethal gene combinations (with *uvrD, rep,* and *radA*, but not *rnhA* or *dam*) were suppressed by deletion of *recF* or *recO*, indicating that their genetic relationships involved roles in post-replication gap repair. Additionally, loss of the RecG/SSB interaction phenocopied a *recG* deletion when combined with *dam*, *uvrD*, *radA*, or *rnhA* deletions but not with *rep*. The results reinforce the idea of RecG as a general genome guardian. RecG has at least two functions. It plays an important role in the resolution of joint molecules behind the fork, formed during post-replication gap repair. RecG is also required to suppress genome over-replication caused by unscheduled replication initiation at R-loops, at double-strand breaks caused by *dam* inactivation, and during replication termination.

**IMPORTANCE:**

DNA damage and subsequent DNA repair processes are mutagenic in nature and an important driver of evolution in prokaryotes, including antibiotic resistance development. Genetic screening approaches, such as transposon sequencing (Tn-seq), have provided important new insights into gene function and genetic relationships. Here, we employed Tn-seq to gain insight into the function of the *recG* gene, which renders *Escherichia coli* cells moderately sensitive to a variety of DNA-damaging agents when they are absent. The reported *recG* genetic interactions can be used in combination with future screens to aid in a more complete reconstruction of DNA repair pathways in bacteria.

## INTRODUCTION

High-fidelity transmission of genetic information during cell division requires complete and accurate duplication of genomic DNA. However, the DNA replication machinery frequently encounters lesions in template DNA that require repair, often with recombination as a key step (1–3). Such repair pathways are major sources of mutagenesis that can lead to the development of cancer in eukaryotes and antibiotic resistance in prokaryotes. *Escherichia coli* has served as an indispensable model organism for the study of the fundamentals of DNA repair and recombination, establishing features of genome maintenance pathways that are well conserved across all domains of life.

One member of these repair pathways, *recG*, was first identified in a screen for mutations that render *E. coli* recombination deficient (4). The RecG protein is a 76-kDa superfamily (SF) 2 helicase found in almost all bacterial species (5) and has been implicated in several DNA repair roles and pathways. Loss of RecG in *E. coli* results in modest sensitivity to DNA-damaging agents such as mitomycin C, ultraviolet (UV) light, and ionizing radiation (6, 7). Cells lacking *recG* struggle to segregate chromosomes after DNA replication and frequently form filaments (8).

RecG binds and remodels several types of DNA substrates *in vitro*, but its preferred substrates are branched DNAs, such as three-strand structures and Holliday junctions (9, 10). A structure of RecG from *Thermotoga maritima* has been solved bound to a synthetic three-way junction, revealing three domains: a “wedge” domain that is involved in DNA junction binding and unwinding and two RecA-like helicase domains responsible for ATP hydrolysis activity and motor function (11). RecG can also unwind D-loops and R-loops, sites where constitutive stable DNA replication (cSDR) can be initiated (12, 13). *E. coli* strains lacking *recG* display cSDR activity, and loss of *recG* results in overamplification of DNA in the terminus region of the chromosome and around double-strand breaks (DSBs) (14–17). These findings suggest an important role for RecG in preventing a lethal DNA replication cascade resulting from continuous reloading of the DnaB helicase by the replication restart protein PriA (15, 18). This cascade is negated when PriA helicase activity is eliminated (18), and suppressors of the Δ*recG* phenotype often manifest as missense mutations within PriA helicase motifs (19–21). In agreement with these observations, the Δ*recG* phenotype is also suppressed by deletion of the replication restart gene *priB* in strains containing additional mutations in genes encoding major subunits of RNA polymerase (*rpoA*, *rpoB*, or *rpoC*) (22).

RecG interacts with the single-stranded DNA (ssDNA) binding protein (SSB) (23, 24). A recent investigation of the interaction with purified proteins revealed that the interaction is mediated by the highly conserved SSB-Ct interaction motif, which comprises the eight C-terminal residues of SSB (25). The SSB-Ct docks onto a highly conserved surface on the RecG helicase domain 2, framed by residues Arg474, Arg614, and Arg467. Perturbations of any of the three Arg residues result in loss of interaction with SSB and partial loss of RecG function *in vivo*, as demonstrated by cell UV sensitivity, SOS induction, and filamentation (25).

Genetic interaction between *recG* and the *radD* (formerly *yejH*) gene has been explored (19, 20). RadD is a putative SF2 helicase and is important for *E. coli* survival after ionizing radiation treatment (7) and for accelerating the rate of RecA-mediated strand exchange *in vitro* (26). Deletion of both *recG* and *radD* results in a severe growth defect that leads to the formation of spontaneous suppressor mutations in *priA* helicase motifs and the *recA* promoter (19). The Δ*recG*Δ*radD* phenotype is also suppressed by deletion of either the recombination mediator *recF* or *recO*, both of which encode proteins that help load RecA onto SSB-coated ssDNA gaps to promote recombination. Characterization of the *recG*-*radD* interaction provides further evidence for a role for both proteins in alleviating toxic DNA situations, likely resulting from an accumulation of recombination intermediates.

Several additional genetic interactions between *recG* and DNA metabolism genes have been identified. Synthetic lethality has been reported for strains lacking *recG* and *uvrD* (27), *rnhA* (14, 18), *polA* (14, 28), or *priA* (29). Similar to a Δ*recG* Δ*radD* strain, Δ*recG* Δ*uvrD* cells experience a “death by recombination” phenotype that can be suppressed by the deletion of *recF* or *recO*, as well as other genes known to be involved in promoting recombination or an accumulation of recombination intermediates, including *recQ* (27). The synthetic lethality of cells lacking *recG* and *rnhA* (which encode RNase HI) likely stems from the toxicity associated with constitutive stable DNA replication, or cSDR (14). RecG is also required for cell viability when 3′ ssDNA exonucleases are absent, and this lethality is a consequence of PriA activity (18). Although PriA helicase activity can be detrimental when *recG* is absent, deletion of *recG* is synthetically lethal with *priA-*null mutations, as PriA is necessary for DnaB loading in the absence of RecG (29).

Strong interactions between *recG* and *ruvABC* (30), *radA* (31, 32), *rarA* (33), and *dam* (28, 34) have also been reported. The synergistic sensitivity of a Δ*recG* Δ*radA* strain to the nucleotide analogue azidothymidine is suppressed by deletion of *recF* (31). The limited viability of *recG ruv* mutant strains improves when mutations are made to *recF*, *recO*, *recR*, *recJ*, or *recQ* (35). Triple deletion of *recG*, *ruvB*, and *rarA* is synthetically lethal, a condition again suppressed by further deletion of *recF* or *recO* (33). Partially overlapping functions in processing Holliday junctions between RecG and RuvAB/C have been well established (9, 30).

The RecF, RecO, and RecR proteins serve to load the RecA protein into post-replication gaps created by replisome lesion-skipping (36). The RecA protein thus loaded generates recombination intermediates, or joint molecules, that link together the two recently replicated chromosome copies. Failure to resolve the joint molecule can result in cell death since the linkage prevents chromosome segregation at cell division (36). If one or more of the RecFOR proteins are missing, the joint molecules are not generated. Thus, deleterious effects that are suppressed by inactivating RecF, RecO, or RecR can be traced to a role in the resolution of joint molecules behind the replication fork, a role that is no longer needed if the joint molecules are not generated.

The previous identification of *recG* genetic interactions summarized above has been driven by targeted approaches involving deletion of *recG* in combination with a second gene of interest to study the resulting phenotype(s). While informative, such approaches are not systematic and have provided a limited scope of the entirety of genetic interactions in *recG*. Previously, we employed transposon sequencing (Tn-seq) (37, 38), an untargeted genetic screening approach, to screen for genes involved in the survival of ionizing radiation in *E. coli* and to link a DNA replication restart pathway to DSB repair (7, 39). Here, we used Tn-seq to systematically evaluate the presence and relative strength of interactions between *recG* and every known nonessential gene in *E. coli*. We identify genes that become conditionally essential or conditionally important in the absence of the *recG* gene, confirm several of these genetic interactions with additional methods, evaluate suppression of synthetic lethality with *recF* or *recO* deletions, and test whether loss of the interaction between RecG and SSB contributes to lethality.

## MATERIALS AND METHODS

### Bacterial strains and primers

Strains used in this study are *E. coli* K12 MG1655 and its derivatives (Table 1). Chromosomal gene knockouts were generated by λ-Red recombination and/or added to strains by P1 transduction, as previously described (19, 40). Deletion of *recG* was added to strains after transformation of each strain with pJJ100, which harbors a wild-type copy of *recG*. Deletions of *recO* or *recF* were added last to strains due to the impaired recombination ability of the resulting strains. PAGE-purified primers were purchased from Integrated DNA Technologies (Table 2), and terminal phosphorothioate bonds are denoted with an asterisk.

**TABLE 1:** Strains used in this study

Strain	Relevant genotype	Parent strain	Source/technique
MG1655	*recG* ^+^		
EAW114	∆*recO*	MG1655	λ-Red recombination
EAW252	*Founder* Δ*e14* Δ*radA*		λ-Red recombination
EAW325	*Founder* Δ*e14* Δ*rep*		λ-Red recombination
EAW408	∆*lacIZYA*	MG1655	λ-Red recombination
EAW505	∆*recG*	MG1655	λ-Red recombination
EAW583	*Founder* Δ*e14* Δ*uvrD*		λ-Red recombination
EAW629	∆*recF*	MG1655	λ-Red recombination
EAW1102	∆*recG* Δ*lacIZYA*/ pJJ100	EAW408	λ-Red recombination
EAW1435	Δ*dam* Δ*lacIZYA*	EAW408	λ-Red recombination
EAW1574	Δ*recG* Δ*rep* Δ*recO* Δ*lacIZYA*/ pJJ100	NJB01/pJJ100	P1 grown on EAW114
EAW1575	Δ*recG* Δ*radA* Δ*recO* Δ*lacIZYA*/ pJJ100	NJB02/pJJ100	P1 grown on EAW114
EAW1577	Δ*recG* Δ*uvrD* Δ*recO* Δ*lacIZYA*/ pJJ100	NJB12/pJJ100	P1 grown on EAW114
EAW1578	Δ*recG* Δ*dam* Δ*recO* Δ*lacIZYA*/ pJJ100	EAW1435/pJJ100	P1 grown on EAW114
EAW1650	*recG-R474E*	MG1655	λ-Red recombination
EAW1693	*recG-R614E*	MG1655	λ-Red recombination
EAW1707	*recG-R484E*	MG1655	λ-Red recombination
NJB01	∆*rep* ∆*lacIZYA*	ΕΑW408	P1 grown on EAW325
NJB02	∆*radA* ∆*lacIZYA*	ΕΑW408	P1 grown on EAW252
NJB03	∆*rnhA* ∆*lacIZYA*	ΕΑW408	P1 grown on Keio Δ*rnhA*
NJB10	∆*recG* ∆*rep* ∆*lacIZYA/* pJJ100	NJB01/pJJ100	P1 grown on EAW505
NJB11	∆*recG* ∆*radA* ∆*lacIZYA*/ pJJ100	NJB02/pJJ100	P1 grown on EAW505
NJB12	∆*uvrD* ∆*lacIZYA*	EAW408	P1 grown on EAW583
NJB13	∆*recG* ∆*rnhA* ∆*lacIZYA*/ pJJ100	NJB03/pJJ100	P1 grown on EAW505
NJB18	∆*recG* ∆*uvrD* ∆*lacIZYA*/ pJJ100	NJB12/pJJ100	P1 grown on EAW505
NJB32	Δ*recG* Δ*dam* Δ*lacIZYA*/ pJJ100	EAW1435/pJJ100	P1 grown on EAW505
NJB62	Δ*rep* Δ*lacIZYA recG-R474E*/ pJJ100	NJB01/pJJ100	P1 grown on EAW1650
NJB63	Δ*radA* Δ*lacIZYA recG-R474E*/ pJJ100	NJB02/pJJ100	P1 grown on EAW1650
NJB64	Δ*rnhA* Δ*lacIZYA recG-R474E*/ pJJ100	NJB03/pJJ100	P1 grown on EAW1650
NJB65	Δ*uvrD* Δ*lacIZYA recG-R474E*/ pJJ100	NJB12/pJJ100	P1 grown on EAW1650
NJB66	Δ*dam* Δ*lacIZYA recG-R474E*/ pJJ100	EAW1435/pJJ100	P1 grown on EAW1650
NJB67	Δ*recG* Δ*rep* Δ*recF* Δ*lacIZYA*/ pJJ100	NJB01/pJJ100	P1 grown on EAW629
NJB68	Δ*recG* Δ*radA* Δ*recF* Δ*lacIZYA*/ pJJ100	NJB02/pJJ100	P1 grown on EAW629
NJB69	Δ*recG* Δ*uvrD* Δ*recF* Δ*lacIZYA*/ pJJ100	NJB12/pJJ100	P1 grown on EAW629
NJB70	Δ*recG* Δ*dam* Δ*recF* Δ*lacIZYA*/ pJJ100	EAW1435/pJJ100	P1 grown on EAW629
NJB71	*recG-R474E* Δ*lacIZYA*	EAW408	P1 grown on EAW1650
NJB72	Δ*rep* Δ*lacIZYA recG-R484E*/ pJJ100	NJB01/pJJ100	P1 grown on EAW1707
NJB73	Δ*radA* Δ*lacIZYA recG-R484E*/ pJJ100	NJB02/pJJ100	P1 grown on EAW1707
NJB74	Δ*rnhA* Δ*lacIZYA recG-R484E*/ pJJ100	NJB03/pJJ100	P1 grown on EAW1707
NJB75	Δ*uvrD* Δ*lacIZYA recG-R484E*/ pJJ100	NJB12/pJJ100	P1 grown on EAW1707
NJB76	Δ*dam* Δ*lacIZYA recG-R484E*/ pJJ100	EAW1435/pJJ100	P1 grown on EAW1707
NJB80	*recG-R484E* Δ*lacIZYA*	EAW408	P1 grown on EAW1707

**TABLE 2:** Oligonucleotides used in this study

Name	Sequence		PAGE purified
Tn5 Amp	5′	CTG TCT CTT ATA CAC ATC TC	✓
Tn-enrich	5′	AAT GAT ACG GCG ACC ACC GAG ATC TAC ACG CAT GCA AGC TTC AGG GTT GAG ATG TGT ATA AGA GAC A*G	✓
Read 1	5′	CTA CAC GCA TGC AAG CTT CAG GGT TGA GAT GTG TA	✓
Index 1	5′	AGA TCG GAA GAG CGT CGT GTA GGG AAA GAG TGT	✓
AM103	5′	CAA GCA GAA GAC GGC ATA CGA GAT ACA TCG ACA CTC TTT CCC TAC ACG A*C	✓
AM104	5′	CAA GCA GAA GAC GGC ATA CGA GAT TGG TCA ACA CTC TTT CCC TAC ACG A*C	✓
AM105	5′	CAA GCA GAA GAC GGC ATA CGA GAT CAC TGT ACA CTC TTT CCC TAC ACG A*C	✓
AM106	5′	CAA GCA GAA GAC GGC ATA CGA GAT GAT CTG ACA CTC TTT CCC TAC ACG A*C	✓
AM068	5′	CAA GCA GAA GAC GGC ATA CGA GAT ATT GGC ACA CTC TTT CCC TAC ACG A*C	✓
AM069	5′	CAA GCA GAA GAC GGC ATA CGA GAT TAC AAG ACA CTC TTT CCC TAC ACG A*C	✓

### Transposome preparation and transposition reactions

Transposon mutagenesis was performed as previously reported (7), with the following exceptions. The EZ-Tn5 <KAN-2> transposon (Epicentre) was amplified with the oligonucleotide Tn5 Amp and Phusion High-Fidelity Polymerase (New England Biolabs). Tn5 DNA (~2.5 pmol) was incubated with 5 µL of Tnp E54K/M56A/L372P transposase (0.492 mg/mL) (41) at room temperature for 3 h. The reaction was diluted to a final volume of 20 µL in Tris-EDTA buffer.

The preparation of electrocompetent cells for transposition was performed as previously described (7). Briefly, cells were cultured in 1 L of Super Optimal broth with Catabolite repression (SOC) medium (2% tryptone, 0.5% yeast extract, 10 mM NaCl, 2.5 mM KCl, 10 mM MgCl_2_, 20 mM glucose) at 37°C with shaking to an OD_600 nm_ of 0.4 to 0.6, chilled at 4°C, pelleted, and washed three times with cold 10% glycerol. Cells were resuspended in 3 mL of cold glycerol-yeast extract medium [10% glycerol (vol/vol), 0.125% yeast extract, 0.25% tryptone] and stored at −80°C. One hundred microliter aliquots of cell suspension were mixed with 5 µL of transposome and electroporated at 2.5 kV. Cells were recovered in 1 mL of SOC medium at 37°C for 1 h, spread on multiple 15 mm by 150 mm Super Optimal Broth (2% tryptone, 0.5% yeast extract, 10 mM NaCl, 2.5 mM KCl, 10 mM MgCl_2_, 10 mM MgSO_4_) round plates supplemented with 40 µg/mL kanamycin, and incubated overnight. Total colony counts were estimated by counting the number of colonies on a one-third section of each plate. Colonies were pooled in Luria Broth (LB) and stored with 10% dimethyl sulphoxide at −80°C. Transpositions were repeated to create triplicate pools of transposon insertion mutants in an MG1655 (wild-type) strain and an MG1655 Δ*recG* (EAW505) strain, with completed libraries totaling an estimated of >450,000 mutants per strain.

### Library sample preparation, sequencing, and data analysis

Each Tn-seq library replicate was used to inoculate 100 mL of LB to an OD_600 nm_ of 0.02 and grow overnight at 37°C with shaking. Genomic DNA was isolated from each culture (Promega Wizard gDNA extraction kit) and mechanically sheared to an average size between 400 and 500 bp. The preparation of the fragment library for sequencing was performed as described using the NEBNext Ultra II DNA Library Prep Kit for Illumina (#E7645), USER Enzyme (New England BioLabs), and AxyPrep Mag PCR Clean Up Kit. Custom primers oAM103, oAM104, oAM105, oAM106, oAM068, or oAM069 and Tn-enrich were used for the PCR amplification step so that only sequences flanking transposons were amplified (Table 2). Tn-enrich was the forward primer and is complementary to the adaptor. Custom reverse primers oAM068, -069, and -103–106 contained different indexes (bolded in Table 2) for multiplexing. The amplified DNA fragment libraries were sequenced on a single-end Illumina NextSeq full flowcell for 75 cycles with 25% PhiX DNA and primers Read 1 and Index 1.

Data were analyzed using a previously described method (7, 39, 42) with slight variations. Sequencing data were checked for transposon sequence enrichment in the first 10 bases using the sequence TAAGAGACAG. The number of reads containing the transposon sequence was divided by the entire read count to ensure data quality. The data were analyzed using a two-sample analysis. Five percent of the start and end of each gene was discarded in the analysis, and only insertions with greater than five sequencing reads were counted. Potential screen hits were chosen based on a greater than 4 log_2_-fold decrease in insertion sequencing reads in a particular gene in the Δ*recG* library as compared to the wild-type library. Genes with significant *P*-values (<0.05) for log-fold decreases in read count or number of insertions were sorted and chosen for further validation.

### Mini-F synthetic lethality assay

The pJJ100 plasmid is a *recG*^+^ derivative of the mini-F low-copy *lac*^+^ pRC7 plasmid and was a generous gift from Christian Rudolph, constructed as previously described (43, 44). The synthetic lethality assay was performed as previously described (19). Briefly, strains were transformed with pJJ100 before the chromosomal *recG* deletion was added. Cultures of each strain with the pJJ100 plasmid and 50 µg/mL ampicillin were grown overnight. The following day, 5 mL of LB without antibiotic was inoculated with a 50-µL overnight culture. Cultures were grown to an OD_600 nm_ of 0.2, immediately serially diluted in 1× phosphate buffered saline (PBS) buffer (137 mM NaCl, 2.7 mM KCl, 10 mM Na_2_HPO_4_, 1.8 mM KH_2_PO_4_, 1 mM CaCl_2_, and 0.5 mM MgCl_2_), and spread on LB plates supplemented with 500 µM isopropyl β-D-1-thiogalactopyranoside (IPTG) and 60 µg/mL X-gal (stored in the dark). Plates were incubated at 37°C for 16–18 h before blue and white colonies were counted, and plates were photographed. All experiments were repeated in biological triplicate with similar results. Bar graphs were generated in GraphPad Prism, where statistical tests (Brown-Forsythe and Welch analysis of variance) were conducted to determine significance.

### DNA damage sensitivity assays

Strains MG1655, Δ*recG*, *recG-R474E*, *recG-R614E*, and *recG-R484E* were grown overnight. Saturated overnight cultures were diluted 1:100 in fresh LB, grown to an OD_600 nm_ of 0.2, and immediately serially diluted in 1× PBS. Ten microliters of each dilution was spotted onto square LB plates supplemented with ciprofloxacin, mitomycin C, hydroxyurea, trimethoprim, or nitrofurazone. Once the spots had adsorbed into the plates, the plates were incubated at 37°C overnight under foil and photographed on an iBright CL1000 (Thermo Fisher Scientific) imaging system the following morning. The plates were poured fresh the day of the experiment and kept under foil to avoid degradation of the DNA-damaging agents.

### Growth competition assay

Strain fitness was assessed using a modified growth competition assay (45, 46) described previously (25). Briefly, saturated cultures of Δ*araBAD* and *araBAD*^+^ strains were mixed at equal volumes and used as inoculum (1:100) into fresh LB. Mixed cultures were grown at 37°C with shaking and reinoculated into fresh medium every 24 h. At 0, 24, 48, and 72 h of incubation, cultures were serially diluted in 1 × PBS and spread on tetrazolium arabinose plates (1% tryptone, 0.1% yeast extract, 0.5% NaCl, 1% arabinose, 0.005% 2,3,5-triphenyltetrazolium chloride, 1.6% agar). Plates were incubated at 37°C overnight before counting white (*araBAD*^+^) and red (Δ*araBAD*) colonies. Growth competition graphs represent data from biological triplicate experiments, with the average percentage of red and white colonies reported. One standard deviation from the mean is represented by error bars.

## RESULTS

### Tn-seq is performed to identify *recG* genetic interactions

**FIG 1:**
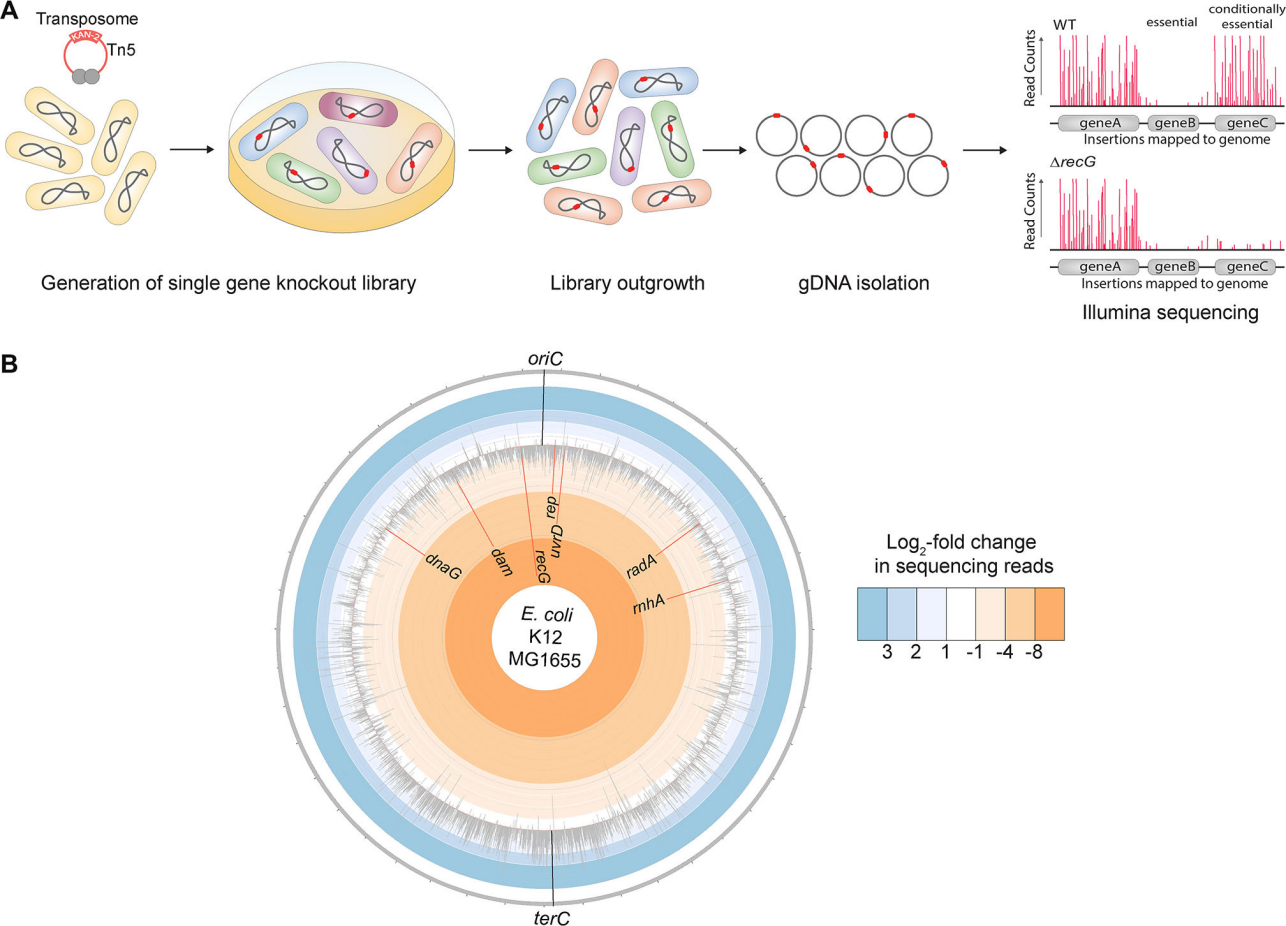
Tn-seq schematic and overview of insertion changes across the *E. coli* genome. (**A**) Schematic showing the general process used to carry out the Tn-seq screen, which involved generating single gene knockout libraries in wild-type and Δ*recG* cells, growing libraries, gDNA isolation of the cultures, and Illumina sequencing. An example of the insertion profiles expected for nonessential, essential, and conditionally essential genes is drawn. (**B**) Circos plot showing the log_2_-fold change in sequencing reads between wild-type and Δ*recG* libraries for all genes in *E. coli* (light gray) and top screen hits (red). The positions of *oriC* and *terC* are marked in black to orient them to the chromosome.

Tn-seq libraries consisting of more than 450,000 insertion mutants were generated in *E. coli* K12 MG1655 (wild-type) and an MG1655 derivative lacking the *recG* gene (EAW505) (Fig. 1). Transformants were pooled together in groups of ~150,000 to form three library replicates per strain. Each pooled library replicate was subjected to a competitive outgrowth phase. Genomic DNA was extracted and prepared for sequencing using commercially available kits, and the prepared libraries were sequenced using Illumina Next Generation Sequencing technology.

Each library replicate generated 20 to 30 million sequencing reads, with the total reads per library amounting to over 75 million reads (Table 3). Over 97% of sequencing reads contained perfect barcode matches, and over 85% of sequencing reads contained the transposon sequence (Table 3). An average of one insertion every 24–25 bases per replicate was calculated for each sample, suggesting a high-density Tn-seq library.

**TABLE 3:** General sequencing results

Strain (replicate)	Total no. of reads	% of reads with perfect barcode	No. of reads containing Tn5 (% of raw reads)	Avg no. of unique insert sites (>5 reads)	Avg distance between inserts (bp)
Wild-type (1)	32,389,930	98.69	27,926,030 (86.2)	190,246	24.4
Wild-type (2)	35,298,206	97.51	31,961,482 (90.5)		
Wild-type (3)	37,051,316	98.92	33,314,880 (89.9)		
Δ*recG* (1)	25,850,011	98.71	22,053,396 (85.3)	189,256	24.5
Δ*recG* (2)	22,987,881	98.39	19,919,770 (86.6)		
Δ*recG* (3)	27,593,556	98.78	24,702,866 (89.5)		

Transposon insertion sites were evaluated in parallel between the wild-type and Δ*recG* libraries. Parallel sequencing results revealed genes that, when disrupted by transposon insertions in the absence of *recG*, caused cells to become inviable or less viable than when disrupted in the wild-type strain. The number of transposon insertion sequencing reads per gene in each library was evaluated and is represented in Fig. 1B across the *E. coli* chromosome as a log_2_-fold change. Negative log-fold change values represent genes in which transposon insertion reads mapped less frequently in the Δ*recG* library than in the wild-type library, and positive log-fold change values represent the opposite effect.

Genes with the most negative log-fold changes in insertion sequencing reads are considered the strongest screen “hits.” The gene with the most negative log-fold change in sequencing reads (−11.2) was *recG*, which is due to its presence and tolerance to disruption in the wild-type strain and its removal in the Δ*recG* strain. To evaluate genetic interactions with nonessential genes only, genes with less than 12 average unique insertions across replicates were deemed essential and excluded from further evaluation. This threshold identified 549 genes out of 4,314 genes (12.7%) as essential (Table S1). Several methods have been employed to evaluate gene essentiality in *E. coli* with varying results (47, 48). Factors such as medium and growth conditions, the presence and duration of competitive outgrowth, and an experimental approach impact essentiality. Transposon insertion studies typically overestimate the number of essential genes because library outgrowth can eliminate slow-growing mutants from the population (7, 47, 49). However, transposon insertions can also be detected in essential genes, especially in regions such as protein termini or flexible interdomain regions that allow a partially functional protein to be produced (50).

**FIG 2:**
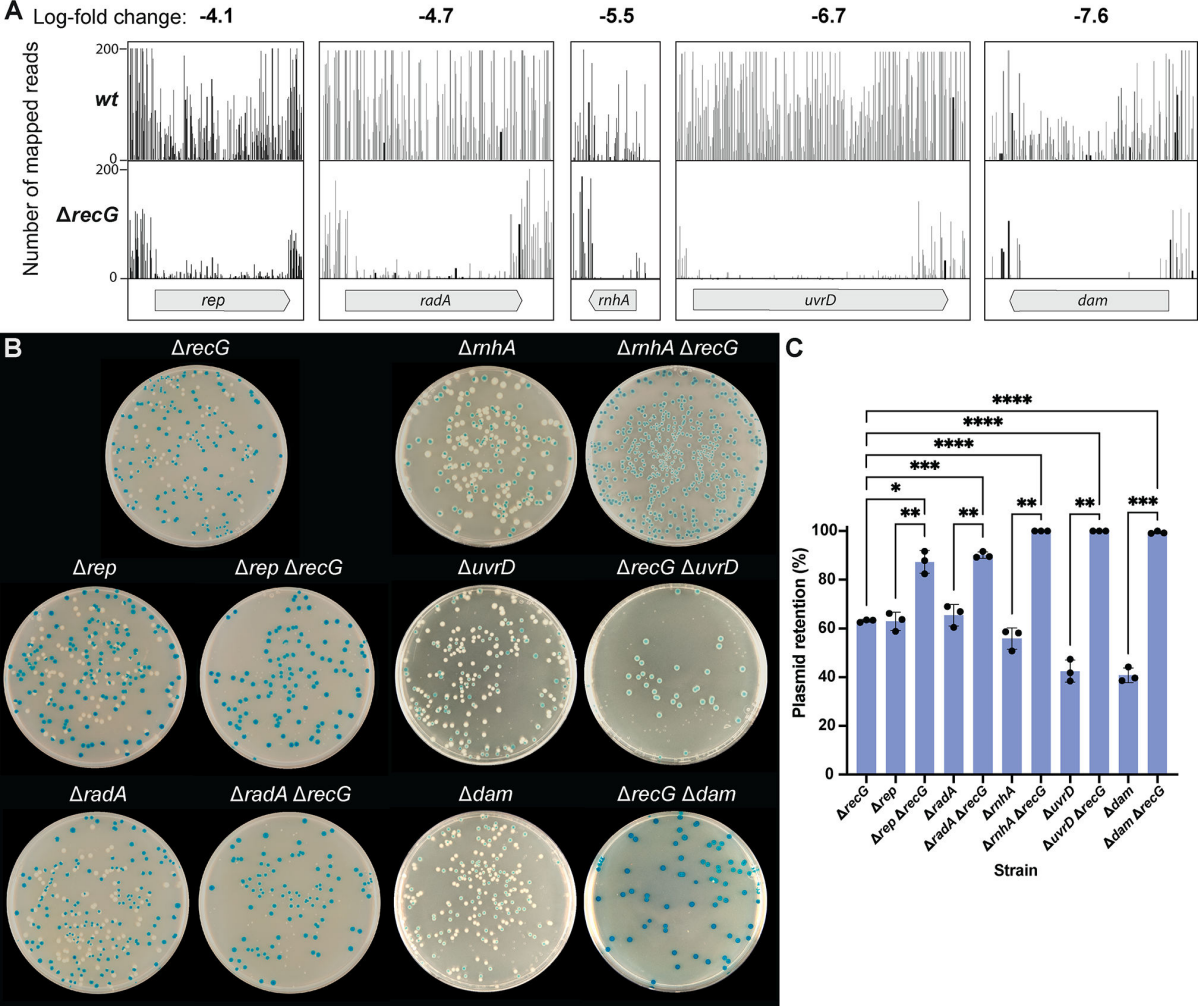
Top five screen hits: *rep*, *radA*, *rnhA*, *uvrD*, and *dam* genes. (**A**) Insertion profiles and the log_2_-fold change in sequencing reads between wild-type and Δ*recG* screens in the genes *rep*, *radA*, *rnhA*, *uvrD*, and *dam*. (Β) Plasmid-based validation assay results confirm synthetic lethal or strong interactions between top screen hits and Δ*recG*. (**C**) Quantification of plasmid retention rate (%) by strain. *, *P* < 0.05; **, *P* < 0.01; ***, *P* < 0.001; ****, *P* < 0.0001. All experiments were performed in triplicate. Bar graphs plot the average value, with error bars representing one SD from the mean.

Of the 3,765 genes deemed nonessential, the largest log-fold changes in transposon insertion sequencing reads were observed in *dam* (−7.6, DNA adenine methyltransferase), *uvrD* (−6.7, DNA helicase), *rnhA* (−5.5, ribonuclease HI), *radA* (−4.7, DNA recombination protein), and *rep* (−4.1, DNA helicase). The presence of insertions in these five genes in the wild-type library population indicates that the genes are nonessential under these conditions and in this strain (Fig. 2A). However, the substantial decrease in both insertion sites and insertion sequencing reads across these five genes in the Δ*recG* background indicates that disruptions to any of these genes result in cells with greatly reduced viability. Such strong effects suggest potential synthetic lethal interactions between the identified genes and *recG*. Synthetic lethality has been previously reported for *recG dam*, *recG uvrD*, and *recG rnhA* double deletion mutants (18, 27, 28), and a strong synergistic effect has been observed for cells lacking both *recG* and *radA* (31). Finally, *rep* represents a new *recG* genetic interaction.

Other genes have been implicated in performing distinct but partially overlapping functions as *recG*, including the *radD* (19), *rarA* (33), and *ruvABC* (33) genes. The log-fold changes for *radD* and *rarA* in the *recG*-deletion strain were −2.2 and −1.4, while the log-fold changes for *ruvABC* were −0.5, −1.4, and −0.3, respectively. Of these genetic interactions, the interaction between *radD* and *recG* is the strongest; viability in double deletion mutants is greatly reduced and only sustained for multiple generations with the accumulation of suppressor mutations (19). Cells lacking *recG* and *rarA* or *recG* and *ruvB* have synergistic sensitivity to DNA-damaging agents (33), but these strains are viable. However, deletion of *recG*, *rarA*, and *ruvB* together is not possible (33). Transposon insertion profiles for *radD*, *rarA*, and *ruvABC* support these previously reported observations (Fig. S1), where insertions are greatly reduced in *radD* when *recG* is absent and more modestly reduced in the *rarA* and *ruvABC* genes.

Additional modest *recG* interactions have been reported, including with the *uvrA* and *recD* genes (6), where double deletion mutants have reduced viability or increased sensitivity to DNA damage. Transposon insertion profiles show that transposon insertions in *uvrA* and *recD* are still well tolerated in cells lacking *recG*, but strong reductions in the number of sequencing reads are observed. The log-fold changes for these genes in the *recG*-deletion strain were −1.6 and −0.8, respectively. This is consistent with double deletion mutants being viable but displaying reduced fitness. Indeed, Δ*recG* Δ*uvrA* and Δ*recG* Δ*recD* strains are outcompeted by a Δ*recG* strain (Fig. S2), confirming fitness defects for the double mutants. The genes with log-fold decreases in sequencing reads between *dam* and *uvrA* are listed in Table S2, and going forward, we will focus on the five strongest screen hits (*dam*, *uvrD*, *rnhA*, *radD*, and *rep*).

### Validation of *recG* synthetic lethal interactions

To independently validate and examine the strongest Tn-seq screen hits, a plasmid-based lethality assay was used. *recG* under the control of its native promoter cloned into the unstable mini-F *lacZ*^+^ pRC7 plasmid (plasmid pJJ100, a kind gift from Christian Rudolph) (19, 44) was transformed into Δ*lacIZYA* strains also lacking *dam*, *uvrD*, *rnhA*, *radD*, or *rep*. Retention of the *recG*^+^ plasmid was evaluated in cultures grown with antibiotic selection pressure withheld. Cultures were plated on LB plates supplemented with IPTG and X-gal, which allowed colonies that retained the mini-F *lacZ*^+^ plasmid (blue) to be distinguished from colonies that lost the plasmid (white). Each of these strains (which had the wild-type chromosomal *recG* gene) readily lost the plasmid-encoded copy of *recG* (Fig. 2B and C). When the plasmid was present in a Δ*recG* background (EAW1102), the plasmid was lost at a similarly high rate (37%; Fig. 2C). This is identical to a result seen previously for this strain (19). The loss of the *recG*-expressing plasmid was somewhat higher (49.8%) when it was introduced into a Δ*lacIZYA* strain with an otherwise wild-type background (EAW408; *recG*^+^), using this same protocol (data not shown).

To evaluate synthetic lethality of double deletion strains in the Δ*lacIZYA* background, *recG* was subsequently deleted from the transformed strains lacking *dam*, *uvrD*, *rnhA*, *radA*, or *rep*. The resulting new set of strains was tested in the plasmid retention assay. Unlike the initial set of strains, which readily lost the *recG*^+^ plasmid, deletion of *recG* in strains already lacking *dam*, *uvrD*, *rnhA*, *radA*, or *rep* resulted in significantly higher rates of plasmid retention (Fig. 2B and C). For the Δ*recG* Δ*rep* and Δ*recG* Δ*radA* strains, a small percentage of white colonies were observed. These white colonies appeared much smaller in size than the blue colonies. Attempts to re-streak these small white colonies resulted in streak plates with no growth or the growth of more small colonies, ruling out the possibility that these colonies are a result of suppressor mutations. Altogether, these results indicate that *recG* is synthetically lethal with *dam*, *uvrD*, and *rnhA*. Furthermore, *recG* is conditionally important but not quite synthetically lethal in cells lacking *radA* and *rep*.

### Suppression by deletions of *recF* or *recO*

Suppression of the synthetic lethality or synergistic sensitivity phenotypes of *recG* and other genes (*radD*, *ruvB*, and *rarA*) by loss of *recF* or *recO* has been reported (19, 33). This is thought to be the case because the RecFOR pathway results in branched DNA intermediate formation in post-replication gaps that utilizes RecG (and other proteins) for processing. To evaluate whether deletion of *recF* or *recO* restores the viability of Δ*recG* Δ*dam*, Δ*recG* Δ*uvrD*, Δ*recG* Δ*rnhA*, Δ*recG* Δ*radA*, and Δ*recG* Δ*rep* strains, the plasmid retention assay was repeated with sets of strains also lacking *recF* or *recO*. We note that, despite several attempts, we were not able to construct Δ*recG* Δ*rnhA* Δ*recF* or Δ*recG* Δ*rnhA* Δ*recO* strains.

**FIG 3:**
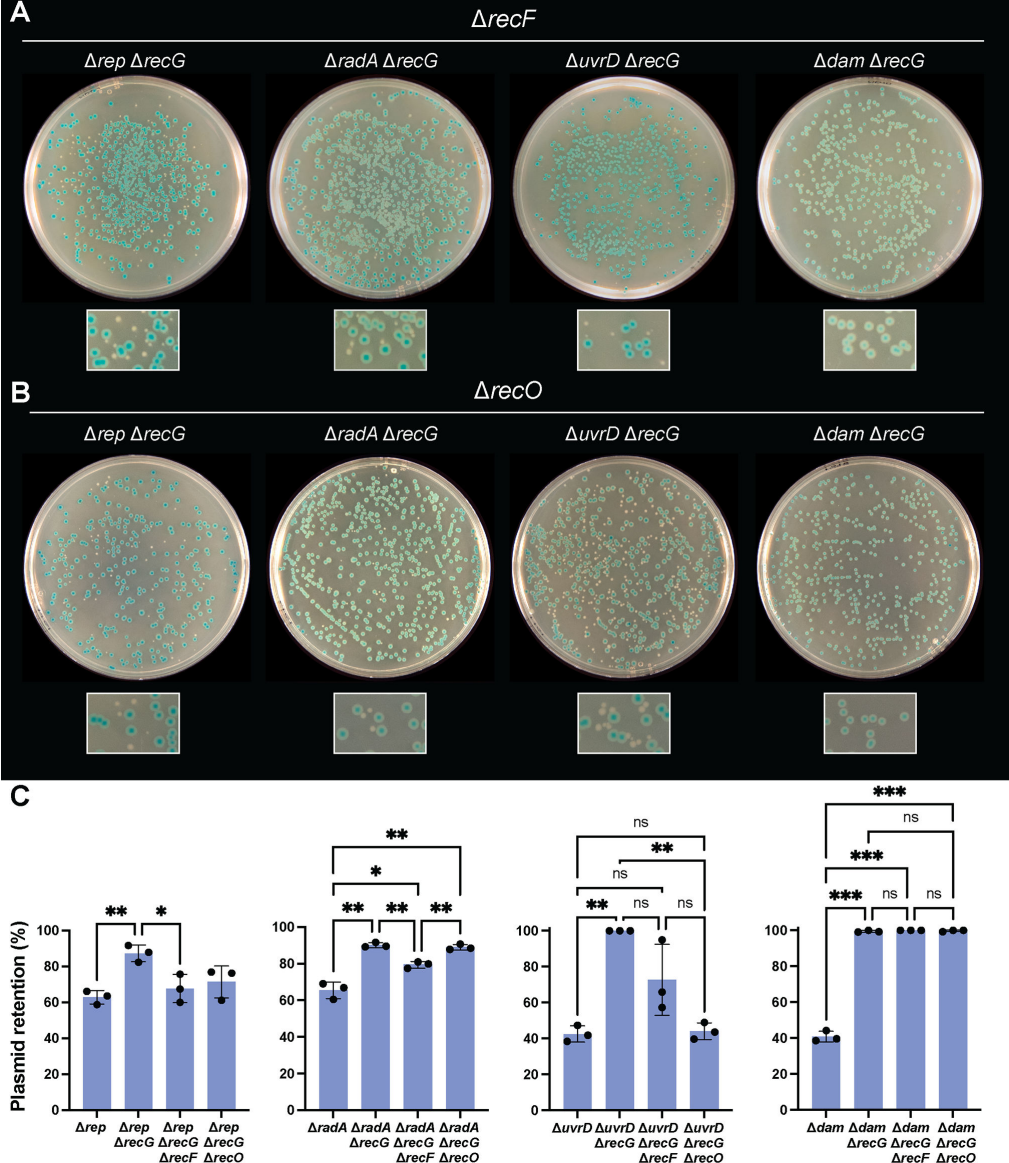
Suppression of synthetic lethality by deletion of *recF* or *recO*. Representative photos of plasmid retention assay blue and white colonies with strains lacking *recF* (**A**) or *recO* (**B**). Small regions of each plate are shown at 2.5× zoom to highlight the presence of small, white colonies. (**C**) Quantification of plasmid retention in strains from assays shown in panels A and B, with bar graphs displaying the mean and error bars representing one SD away from the mean. All experiments were performed in triplicate. *P*-value designations (*, **, and ***) are the same as listed in Fig. 2 legend.

In agreement with previous observations, loss of *recF* or *recO* restores the viability of Δ*recG* Δ*uvrD* cells (Fig. 3), which have been reported to experience a death by recombination (27). However, there do appear to be slight differences in the extent of suppression between a *recF* or *recO* deletion in Δ*recG* Δ*uvrD* cells. Loss of *recO* results in a consistently greater number of white colonies (loss of plasmid) in the plasmid retention assay, and the white colonies appear slightly larger in size than the white colonies that appear in strains lacking *recF*. The opposite effect appears in Δ*recG* Δ*radA* cells. While the white colonies in Δ*recG* Δ*radA* Δ*recF* and Δ*recG* Δ*radA* Δ*recO* experiments appear similar in size, there were consistently more white colonies appearing in the plasmid retention experiments with the Δ*recG* Δ*radA* Δ*recF* strain (Fig. 3). It has been previously reported that loss of *recF* improves the viability of a Δ*recG* Δ*radA* strain (31), but the effect of loss of *recO* has not been reported. These results indicate that loss of *recF* or *recO* does not always produce identical outcomes in people with the same genetic backgrounds. In the case of Δ*recG* Δ*rep* cells, loss of *recF* or *recO* improved the viability of the strain to similar extents.

Interestingly, the synthetic lethality of Δ*recG* Δ*dam* is not suppressed by deletion of *recF* or *recO*, supported by the lack of white colonies present in plasmid retention assays (Fig. 3). This indicates that the mechanism of lethality in Δ*recG* Δ*dam* cells is not RecFOR-dependent.

### Effects of a mutation that abates RecG interaction with SSB

**FIG 4:**
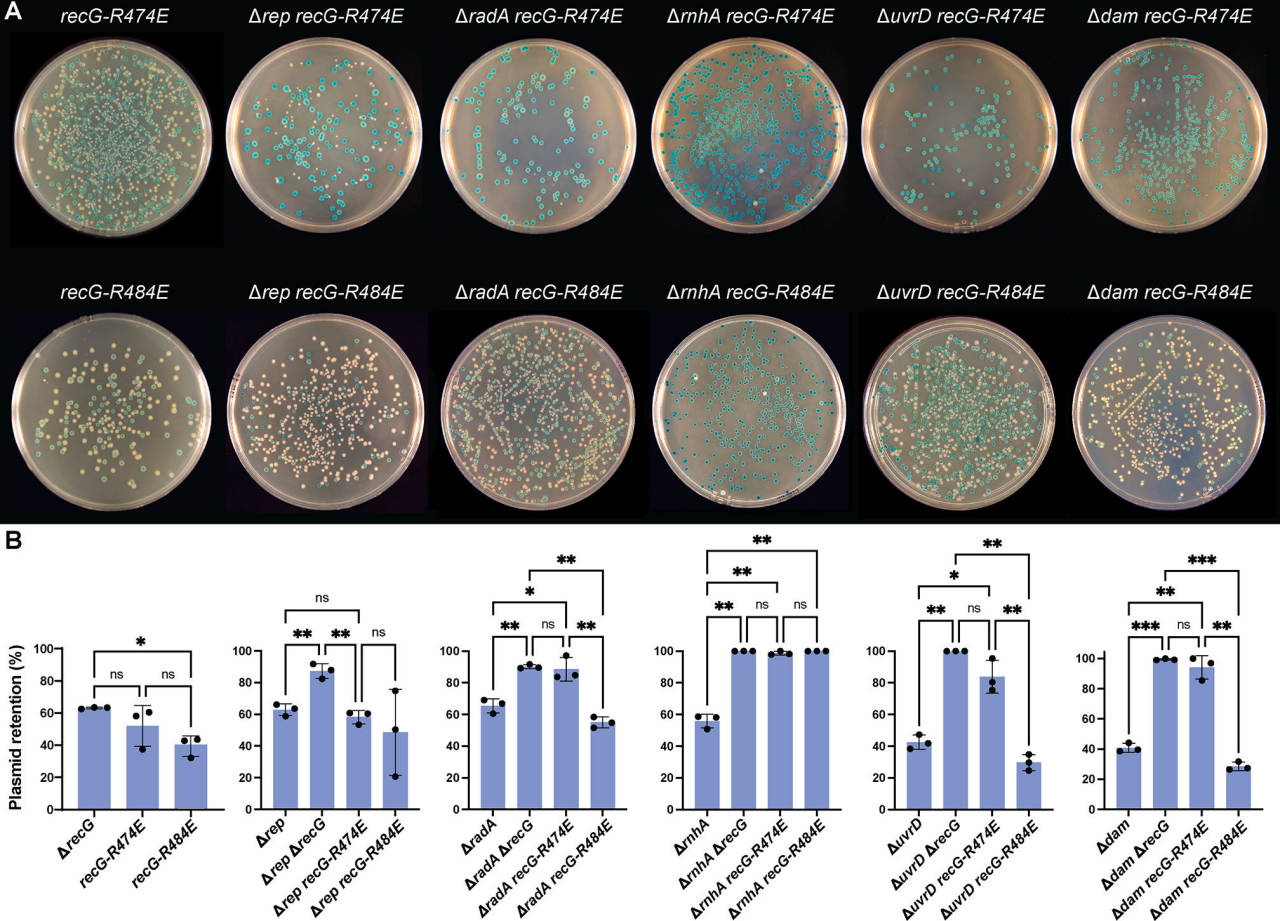
The effects of *recG-R474E* mirror those of Δ*recG* in strains lacking *radA*, *rnhA*, *uvrD*, and *dam*, but not *rep*. (Α) Photos depicting blue and white colonies from plasmid retention assays where deletions in *rep*, *radA*, *rnhA*, *uvrD*, or *dam* are combined with the *recG-R474E* or *recG-R484E* mutation (control). (**B**) Quantification of plasmid retention from experiments depicted in panel A. All experiments were performed in triplicate. Bar graphs plot the mean value, with error bars representing one SD from the mean. *P*-value designations (*, **, and ***) are the same as listed in Fig. 2 legend.

It was recently shown that an interaction with SSB is important for RecG function *in vivo*, with RecG residues R474 and R614 playing essential roles in the interaction between RecG and the SSB C-terminus (21). A *recG-R474E* mutation abates the interaction between RecG and SSB, and a *recG-R474E* strain displays UV sensitivity, increased basal SOS induction, and cell filamentation, albeit not to the same extent as a complete loss of *recG* (25). To test whether the *recG-R474E* mutation produces similar effects to a complete loss of *recG* in the plasmid retention assay, the mutation was added to strains lacking *dam*, *uvrD*, *rnhA*, *radA*, or *rep* in the Δ*lacIZYA* background. As a negative control, we also tested the effects of a *recG-R484E* mutation, which mutates a residue that is near but not in the SSB binding pocket on RecG, in the plasmid retention assay as a negative control. Neither the *recG-R474E* nor the *recG-R484E* mutation alone led to higher levels of *recG*^+^ plasmid retention in *recG^+^*, as expected (Fig. 4).

When combined with deletions in *dam*, *uvrD*, *radA*, or *rnhA*, the *recG-R474E* mutation was synthetically lethal, mirroring the effects of a complete *recG* deletion (Fig. 4). In contrast, loss of the RecG/SSB interaction did not affect Δ*rep* cells, which differed from the synthetic lethality observed with deletion of *recG* in Δ*rep* cells. This difference could help to explain why the *recG-R474E* does not phenocopy Δ*recG* mutation in *E. coli* (21). The results were different for *recG-R484E*. In all cases except for one (*rnhA*), the *recG-R484E* mutation did not impact plasmid retention or strain viability, highlighting the dependence of the genetic interactions on the ability of RecG to interact with SSB.

**FIG 5:**
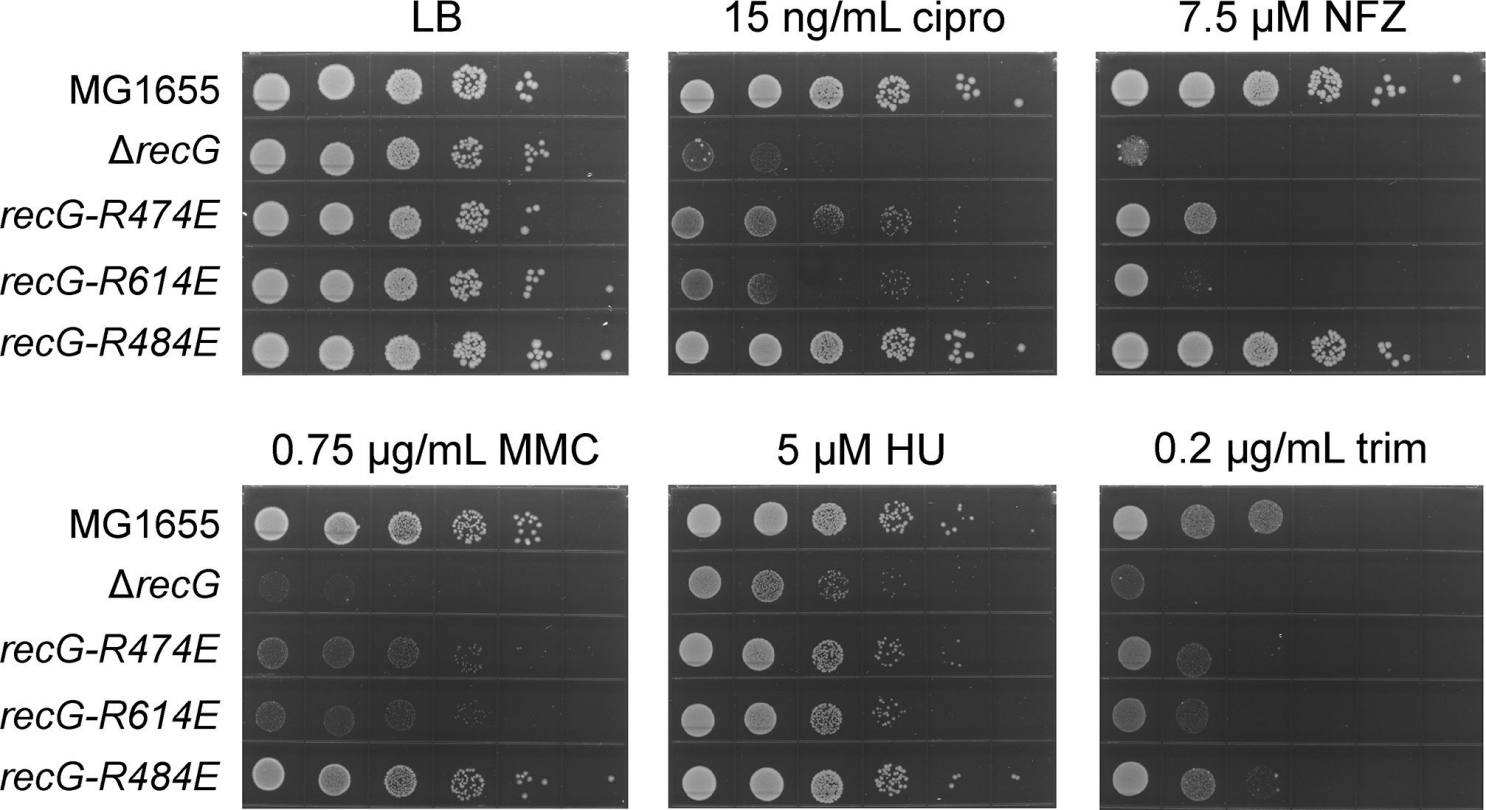
The *recG-R474E* mutant strain displays general DNA damage sensitivity. Strains expressing wild-type *recG* (MG1655), no *recG* (Δ*recG*), a *recG* SSB-binding pocket mutant (*recG-R474E* or *recG-R614E*), or a *recG* HD2 mutant that retains interaction with SSB were evaluated for sensitivity to ciprofloxacin (cipro), nitrofurazone (NFZ), mitomycin C (MMC), hydroxyurea (HU), or trimethoprim (trim). Plating efficiencies on drug plates are compared to those of each strain spotted on LB.

To more broadly examine whether loss of the RecG/SSB interaction produced general or specific DNA damage sensitivity phenotypes, the sensitivity of the *recG-R474E* and *recG-R614E* binding pocket mutant strains to DNA-damaging agents was examined alongside the wild-type (MG1655), Δ*recG*, and *recG-R484E* control strains. *recG-R614E* encodes a RecG variant that is also incapable of SSB binding, and both *recG-R474E* and *recG-R614E* mutant strains are sensitive to UV exposure (25). We found that the *recG-R474E* and *recG-R614E* mutations render cells sensitive to a variety of DNA-damaging agents, including ciprofloxacin, hydroxyurea, trimethoprim, mitomycin C, and nitrofurazone, but the sensitivity was not to the same extent as complete loss of *recG* (Fig. 5).

Altogether, combining the *recG-R474E* mutation with deletions in *dam*, *uvrD*, *rnhA*, *radD*, or *rep* and surveying the sensitivity of the mutant to a panel of DNA-damaging agents reveal a more general role in repair and recombination for the RecG/SSB interaction. It does not appear that loss of the interaction affects a specific RecG repair pathway or function. Instead, loss of interaction generally diminishes but does not entirely eliminate the capacity of RecG to access the sites where it is to function.

## DISCUSSION

This study used Tn-seq to identify several *recG* genetic interactions and gain insight into *recG* function in *E. coli*. The strongest interactions identified in the screen were the *dam*, *uvrD*, *rnhA*, *radA*, and *rep* genes. These genes were either synthetically lethal with Δ*recG* or conditionally important in cells lacking *recG*. The screen results were confirmed with a plasmid-based lethality assay. Evidence for strong interactions between *recG* and *dam* (28, 34), *uvrD* (27, 28), *rnhA* (14), and *radA* (31) has appeared previously, while the interaction we identified with *rep* has not been previously reported. The set of genes listed in Table S2 represents a complete accounting of the strongest *recG* interactions.

We also found differing patterns in suppression of synthetic lethality/conditional importance with deletions of *recF* or *recO*. Deletions in *recF* or *recO* improved the viability of *recG uvrD*, *recG radA*, and *recG rep* mutant strains, but no suppression of synthetic lethality was observed for *recG dam* mutants. We were unable to construct a strain lacking the *recG*, *rnhA*, and *recF* or *recO* genes simultaneously, so we were unable to test for *recFO* suppression directly. Loss of *recO* appeared to restore the health of *recG uvrD* to a greater extent than loss of *recF*, based on qualitative colony size observations. In contrast, *recG radA* viability appeared to be more consistently improved by the loss of *recF* over *recO*. Additionally, we evaluated the effect of the *recG-R474E* mutation on gene interactions and found that loss of the RecG/SSB interaction produced similar effects as a complete loss of *recG* when paired with several other gene deletions. Loss of the RecG/SSB interaction appears to produce general effects that affect RecG activity in some but not all repair pathways it is involved with, along with the response to different types of DNA damage. Altogether, the work is consistent with prior studies indicating an important role for RecG in multiple DNA repair pathways. Here, we develop RecG roles in the repair of post-replication gaps and in the suppression of over-replication by aberrant replication initiation.

The absence of *dam*, *uvrD*, *rnhA*, *radA*, or *rep* is associated with an increase in double-stranded breaks, stalled replication forks, and/or an increase in recombination or accumulation of recombination intermediates (31, 39, 51–57). The functions of these genes appear to become especially important, if not essential, when RecG is absent. Interestingly, differences in the mechanisms leading to the conditional importance of these genes in the absence of *recG* are starting to emerge. We have identified that some (*recG rep*, *recG uvrD*, *recG radA*) genetic interactions are suppressed by *recF* or *recO* deletions, while others are not or probably not (*recG dam*, *recG rnhA*), and that *recF* or *recO* deletion can produce minor differences in suppression (in *recG radA* and *recG uvrD* mutants). The suppression by *recF* and *recO* reflects an important role for RecG in the recombinational repair of post-replication gaps (19, 33).

UvrD modulates recombination by dismantling RecA nucleoprotein filaments (54, 58–60). Loss of UvrD results in an accumulation of recombination intermediates that require RecG Holliday junction resolution activity. The toxicity caused by this accumulation of recombination intermediates is negated when recombination is suppressed by the loss of *recF* or *recO* (27). Recombination intermediate accumulation is also likely the cause of Δ*recG* Δ*radA* cell lethality, as RadA has been implicated in performing similar activities to RecG in processing branched DNA intermediates (32). The cause of lethality with *rep*, which encodes a DNA helicase responsible for clearing replication roadblocks, is not immediately clear and warrants further investigation. The suppression of the synthetic lethality of Δ*rep* Δ*recG* strains by deletion of the genes encoding RecF or RecO suggests that Rep may be yet another helicase (along with RadA, RadD, UvrD, RecQ, RuvB) with a role in preventing the formation of or resolving joint molecules in post-replication gaps in a manner that complements the role of RecG.

Unlike *uvrD*, *rep*, and *radA*, loss of *recF* or *recO* did not suppress the lethality of Δ*recG* Δ*dam*, and a *recF* or *recO* deletion could not be introduced to Δ*recG* Δ*rnhA* cells. Dam is responsible for DNA methylation, which helps direct mismatch repair. Loss of *dam* is associated with double-strand breaks, the repair of which could trigger unscheduled DNA replication behind the fork (51, 61). The *dam recG* results suggest that RecG plays an equally essential role in repairing the DSBs associated with *dam* removal. Dam is also implicated in suppressing cSDR originating from R-loops, as are the RecG and RNase HI proteins (62). The overall theme is that RecG appears to be needed to prevent genome over-replication. When Dam is absent, RecG may be required to act at D-loop intermediates generated by double-strand break repair so as to prevent an unscheduled replication restart that would lead to over-replication of segments of the genome. Similarly, when RNase HI, which degrades RNA:DNA hybrids, is absent, RecG R-loop unwinding may be required to prevent inappropriate replication initiation at these sites. Collectively, the results are also consistent with *in vitro* studies on RecG remodeling of branched structures (10, 63) and *in vivo* work showing that DNA amplification or over-replication is a detrimental problem in cells lacking *recG*, especially at sites of DSBs or replication termination (15, 17).

The *in vivo* function of RecG is dependent to a significant degree on its interaction with SSB (25). However, mutations in *recG* that affect the SSB binding pocket produce phenotypes that are not as severe as a complete loss of the gene. This has led us to question whether the interaction with SSB is important only for some, but not all, RecG functions. To answer this question, we combined the SSB-binding-deficient *recG-R474E* mutation with deletions in *dam*, *uvrD*, *rnhA*, *radD*, or *rep* and surveyed the sensitivity of the mutant to a panel of DNA-damaging agents. The *recG-R474E* mutation led to similar synthetic lethality patterns and DNA damage sensitivity as Δ*recG* for *dam*, *uvrD*, *rnhA*, and *radA*, suggesting that RecG/SSB interaction is important for coordinated RecG function with the proteins encoded by these genes. In contrast, the *recG-R474E* mutation did not affect viability in Δ*rep* cells, whereas deleting *recG* from Δ*rep* cells was lethal. This indicates that RecG interaction with SSB is not required for its coordinated activities with Rep.

**FIG 6:**
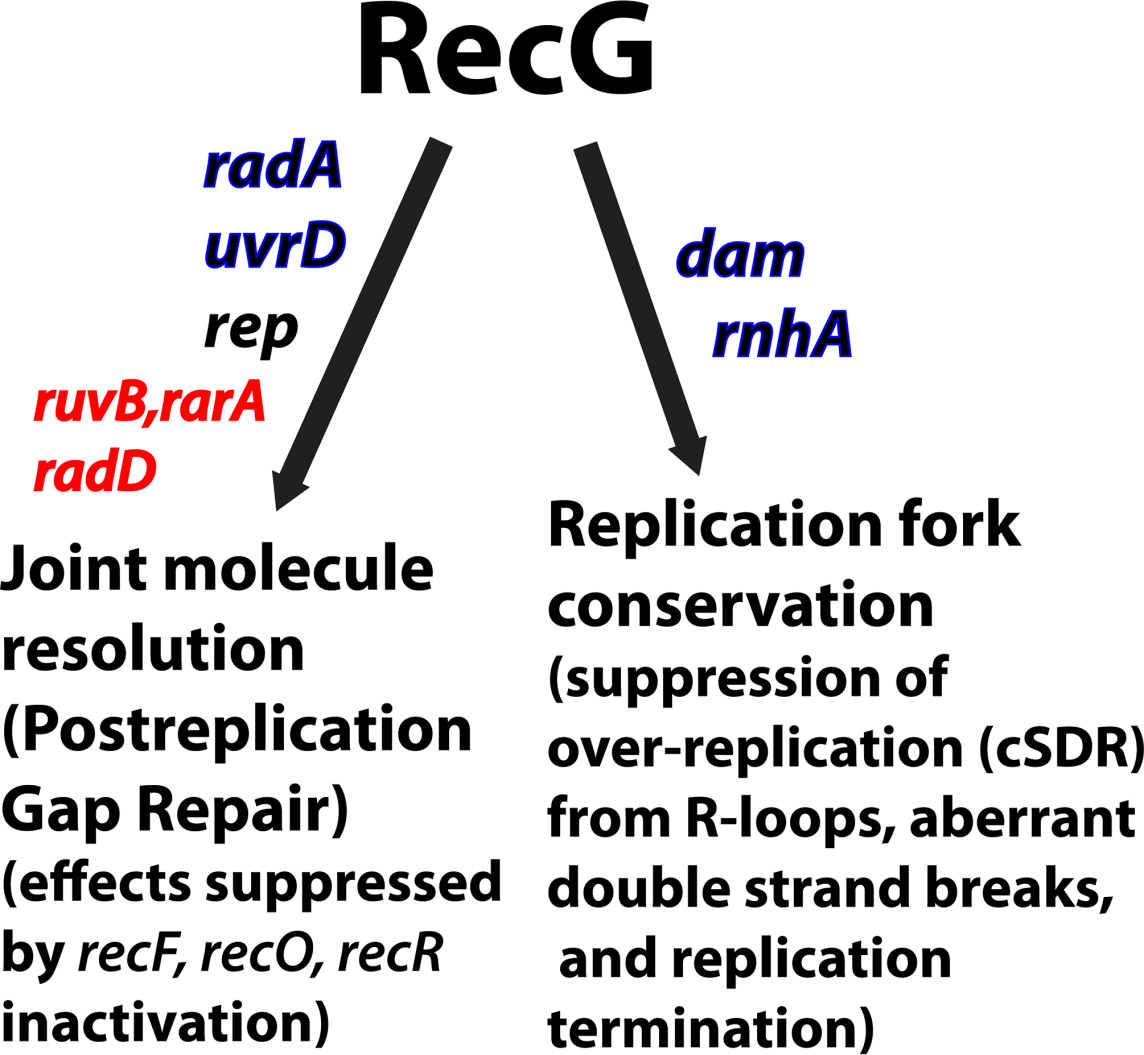
The cellular functions of the RecG helicase. The results of this and previous studies highlight two roles for RecG. The first is in the resolution of recombination intermediates (joint molecules) generated in post-replication gaps behind the replication fork. There are multiple resolution pathways, with RecG important for some but not all. If alternative pathways are blocked, cells die because the replicated chromosomes are linked by joint molecules and cannot be segregated at cell division. Elimination of any of the RecFOR proteins prevents RecA loading and the generation of the joint molecules, alleviating the need for joint molecule resolution and leading to suppression of the deleterious effects of *recG radA*, *recG uvrD*, and *recG rep* double deletions. Additional genetic interactions relevant to post-replication gap repair are indicated in red and discussed in the text. The second role of RecG is suppression of over-replication of genome segments due to unscheduled replication initiation at R-loops, double-strand breaks resulting from *dam* inactivation, or during replication termination. The interaction of RecG with SSB is needed for all RecG functions used to complement the gene inactivations shown in blue.

The results are summarized in Fig. 6. In general, RecG plays important roles in at least two kinds of DNA transactions. Post-replication gap repair, where it plays a role in resolving some, if not many, joint molecules constructed behind the replication fork by RecA, is one. A second involves a role in the suppression of inappropriate replication initiation events at R-loops, double-strand breaks arising from *dam* inactivation, or during replication termination. The function of RecG complements different additional enzymes in these two capacities. However, each role of RecG represents a critical contribution to cell survival. Each separate set of double mutants can result in cell death. Many, but not all, of these transactions require the interaction of RecG with SSB.

We note that while the top five screen hits were the subject of this investigation, Tn-seq allowed us to evaluate the comparative importance of every nonessential gene in wild-type and Δ*recG E. coli*. Genes with known (but weaker) interactions with *recG* (such as *radD*, *ruvABC*, *rarA*, *recD*, *uvrA*) were also observed to be of greater importance in cells lacking *recG* in the Tn-seq data. It is likely that there are additional genes with which *recG* may have a functional relationship identified in the Tn-seq screen (listed in Table S2), and this presents a significant opportunity for future investigation.

Ιn addition to providing insight on *recG* function by studying *recG* genetic interactions and *recG-R474E* phenotypes, we hope that this data set will be useful to the science community for a variety of applications. These applications include evaluating gene essentiality, identifying nonessential domains within essential genes, and identifying regions of genes of interest that may tolerate manipulation, such as the addition of tags or fusion to fluorescent proteins for imaging. The insertion data can also be viewed in programs such as MochiView (64) or Integrative Genomics Viewer (65) to evaluate tolerance of transposon insertions in noncoding or intergenic regions of the genome.

## Data Availability

The raw sequencing data are available at the NCBI SRA under BioProject ID PRJNA976267. Wig files for visualizing insertion profiles in MochiView or Integrative Genomics Viewer are provided at the Dryad repository (https://doi.org/10.5061/dryad.dz08kps2t). The processed sequencing data set Excel file is also provided.

## SUPPLEMENTAL MATERIAL

Tables S1 and S2 and Figures S1 and S2 jb.00184-23-s0001.docx Supplemental tables and figures cited in text.

Processed transposon sequencing dataset jb.00184-23-s0002.xlsx The average unique insertion sites (“hits”) and raw sequencing reads for all genes across three sequenced library replicates for wild-type and Δ*recG* strains. Log-fold changes for average unique insertion sites/sequencing reads, average weighted reads, and *P*-values are also listed by gene ID.

ASM does not own the copyrights to Supplemental Material that may be linked to, or accessed through, an article. The authors have granted ASM a non-exclusive, world-wide license to publish the Supplemental Material files. Please contact the corresponding author directly for reuse.
