# Supplementary material for: Identification of recG genetic interactions in Escherichia coli by transposon sequencing
Source: J Bacteriol. 2023 Nov 29;205(12):e00184-23. doi: 10.1128/jb.00184-23 (PMC10870727; doi:10.1128/jb.00184-23)
Supplement: Tables S1 and S2 and Figures S1 and S2 — Supplemental tables and figures cited in text. [file jb.00184-23-s0001.docx]

**Supplementary Information**

**Supplementary Table 1.** List of genes with <12 unique insertion sites in the wild type library deemed essential

| **Gene ID** | **Gene Name** | **Annotation** | **Ave. Unique hits (control)** |
| --- | --- | --- | --- |
| b0185 | accA | acetyl-CoA carboxylase, carboxytransferase, alpha subunit | 6.67 |
| b3255 | accB | acetyl CoA carboxylase, BCCP subunit | 1.33 |
| b2316 | accD | acetyl-CoA carboxylase, beta (carboxyltransferase) subunit | 7.00 |
| b1094 | acpP | acyl carrier protein (ACP) | 2.33 |
| b2563 | acpS | holo-[acyl-carrier-protein] synthase 1 | 3.33 |
| b0474 | adk | adenylate kinase | 7.00 |
| b3853 | alaT | tRNA-Ala | 8.33 |
| b3276 | alaU | tRNA-Ala | 6.67 |
| b0203 | alaV | tRNA-Ala | 4.00 |
| b2397 | alaW | tRNA-Ala | 0.67 |
| b2396 | alaX | tRNA-Ala | 1.33 |
| b0564 | appY | global transcriptional activator, DLP12 prophage | 7.67 |
| b2691 | argQ | tRNA-Arg | 4.67 |
| b0536 | argU | tRNA-Arg | 3.33 |
| b2694 | argV | tRNA-Arg | 2.33 |
| b2348 | argW | tRNA-Arg | 2.33 |
| b3796 | argX | tRNA-Arg | 0.67 |
| b2693 | argY | tRNA-Arg | 3.00 |
| b2692 | argZ | tRNA-Arg | 4.33 |
| b1166 | ariR | RcsB connector protein for regulation of biofilm and acid-resistance | 7.00 |
| b1977 | asnT | tRNA-Asn | 7.33 |
| b1986 | asnU | tRNA-Asn | 6.67 |
| b1989 | asnV | tRNA-Asn | 7.33 |
| b1984 | asnW | tRNA-Asn | 5.00 |
| b1866 | aspS | aspartyl-tRNA synthetase | 11.67 |
| b3760 | aspT | tRNA-Asp | 3.33 |
| b0206 | aspU | tRNA-Asp | 4.33 |
| b0216 | aspV | tRNA-Asp | 6.67 |
| b3737 | atpE | F0 sector of membrane-bound ATP synthase, subunit c | 5.33 |
| b3736 | atpF | F0 sector of membrane-bound ATP synthase, subunit b | 9.33 |
| b4663 | azuC | acid-inducible small membrane-associated protein | 0.67 |
| b0455 | b0455 | Pseudo gene | 0.67 |
| b1574 | b1574 | Pseudo gene | 3.00 |
| b1954 | b1954 | Pseudo gene | 4.67 |
| b2621 | b2621 | Pseudo gene | 8.33 |
| b2891 | b2891 | peptide chain release factor RF-2 | 10.00 |
| b2911 | b2911 | Pseudo gene | 6.00 |
| b3123 | b3123 | Pseudo gene | 3.67 |
| b3864 | b3864 | Pseudo gene | 4.33 |
| b4047 | b4047 | uncharacterized protein | 3.00 |
| b4414 | b4414 | Pseudo gene | 2.67 |
| b4417 | b4417 | Pseudo gene | 2.67 |
| b4418 | b4418 | Pseudo gene | 8.00 |
| b4420 | b4420 | Pseudo gene | 5.00 |
| b4422 | b4422 | Pseudo gene | 6.33 |
| b4424 | b4424 | Pseudo gene | 6.33 |
| b4425 | b4425 | Pseudo gene | 6.67 |
| b4427 | b4427 | Pseudo gene | 9.00 |
| b4429 | b4429 | Pseudo gene | 3.67 |
| b4430 | b4430 | Pseudo gene | 1.33 |
| b4431 | b4431 | Pseudo gene | 11.67 |
| b4433 | b4433 | Pseudo gene | 4.00 |
| b4437 | b4437 | Pseudo gene | 7.33 |
| b4438 | b4438 | Pseudo gene | 2.67 |
| b4439 | b4439 | Pseudo gene | 4.33 |
| b4441 | b4441 | Pseudo gene | 8.00 |
| b4442 | b4442 | Pseudo gene | 1.67 |
| b4444 | b4444 | Pseudo gene | 10.33 |
| b4445 | b4445 | Pseudo gene | 7.33 |
| b4447 | b4447 | Pseudo gene | 9.33 |
| b4449 | b4449 | Pseudo gene | 8.00 |
| b4450 | b4450 | Pseudo gene | 11.33 |
| b4451 | b4451 | Pseudo gene | 7.00 |
| b4458 | b4458 | Pseudo gene | 6.33 |
| b4585 | b4585 | Pseudo gene | 7.33 |
| b4597 | b4597 | Pseudo gene | 2.67 |
| b4603 | b4603 |  | 1.33 |
| b4608 | b4608 | Pseudo gene | 7.67 |
| b4609 | b4609 | Pseudo gene | 7.67 |
| b4611 | b4611 | Pseudo gene | 11.67 |
| b4616 | b4616 | Pseudo gene | 9.67 |
| b4624 | b4624 | Pseudo gene | 6.67 |
| b4625 | b4625 | Pseudo gene | 9.67 |
| b4690 | b4690 |  | 5.00 |
| b4698 | b4698 | Pseudo gene | 3.67 |
| b4700 | b4700 | Pseudo gene | 5.67 |
| b4701 | b4701 | Pseudo gene | 9.33 |
| b4704 | b4704 | Pseudo gene | 1.67 |
| b4707 | b4707 | Pseudo gene | 9.00 |
| b4712 | b4712 | Pseudo gene | 8.67 |
| b4713 | b4713 | Pseudo gene | 11.67 |
| b2595 | bamD | BamABCDE complex OM biogenesis lipoprotein | 4.00 |
| b1112 | bhsA | biofilm, cell surface and signaling protein | 7.33 |
| b0126 | can | carbonic anhydrase | 4.67 |
| b2198 | ccmD | cytochrome c biogenesis protein, heme export ABC transporter holo-CcmE release factor | 9.00 |
| b0175 | cdsA | CDP-diglyceride synthase | 7.33 |
| b0910 | cmk | cytidylate kinase | 6.00 |
| b3974 | coaA | pantothenate kinase | 8.67 |
| b3634 | coaD | pantetheine-phosphate adenylyltransferase | 8.33 |
| b0103 | coaE | dephospho-CoA kinase | 11.00 |
| b1145 | cohE | e14 prophage, repressor protein phage e14 | 2.33 |
| b1146 | croE | e14 prophage, putative DNA-binding transcriptional regulator | 11.00 |
| b1557 | cspB | Qin prophage, cold shock protein | 8.00 |
| b0623 | cspE | constitutive cold shock family transcription antitermination protein, negative regulator of cspA transcription, RNA melting protein, ssDNA-binding protein | 9.33 |
| b1558 | cspF | Qin prophage, cold shock protein | 6.67 |
| b1552 | cspI | Qin prophage, cold shock protein | 1.33 |
| b2696 | csrA | pleiotropic regulatory protein for carbon source metabolism | 5.00 |
| b0526 | cysS | cysteinyl-tRNA synthetase | 9.67 |
| b1910 | cysT | tRNA-Cys | 1.67 |
| b2478 | dapA | dihydrodipicolinate synthase | 7.00 |
| b0031 | dapB | dihydrodipicolinate reductase | 7.00 |
| b0166 | dapD | 2,3,4,5-tetrahydropyridine-2-carboxylate N-succinyltransferase | 11.67 |
| b2472 | dapE | N-succinyl-diaminopimelate deacylase | 11.00 |
| b3809 | dapF | diaminopimelate epimerase | 9.67 |
| b2065 | dcd | deoxycytidine triphosphate deaminase, dCTP deaminase | 7.33 |
| b3287 | def | peptide deformylase | 8.67 |
| b1570 | dicA | Qin prophage, putative regulator for DicB | 3.33 |
| b1575 | dicB | Qin prophage, cell division inhibition protein | 4.67 |
| b1569 | dicC | Qin prophage, DNA-binding transcriptional regulator for DicB | 5.67 |
| b1061 | dinI | DNA damage-inducible protein I | 11.00 |
| b4613 | dinQ | UV-inducible membrane toxin, DinQ-AgrB type I toxin-antitoxin system | 8.67 |
| b4361 | dnaC | DNA biosynthesis protein | 7.33 |
| b0215 | dnaQ | DNA polymerase III epsilon subunit | 10.33 |
| b4362 | dnaT | DNA biosynthesis protein (primosomal protein I) | 6.33 |
| b3640 | dut | deoxyuridinetriphosphatase | 10.33 |
| b4410 | ecnA | entericidin A membrane lipoprotein, antidote entericidin B | 9.33 |
| b2269 | elaD | protease, capable of cleaving an AMC-ubiquitin model substrate | 10.67 |
| b2779 | eno | enolase | 11.00 |
| b2566 | era | membrane-associated, 16S rRNA-binding GTPase | 7.00 |
| b0156 | erpA | iron-sulfur cluster insertion protein | 6.00 |
| b0954 | fabA | beta-hydroxydecanoyl thioester dehydrase | 6.33 |
| b1092 | fabD | malonyl-CoA-[acyl-carrier-protein] transacylase | 10.00 |
| b1093 | fabG | 3-oxoacyl-[acyl-carrier-protein] reductase | 8.00 |
| b1091 | fabH | 3-oxoacyl-[acyl-carrier-protein] synthase III | 7.00 |
| b1288 | fabI | enoyl-[acyl-carrier-protein] reductase, NADH-dependent | 8.67 |
| b0180 | fabZ | (3R)-hydroxymyristol acyl carrier protein dehydratase | 3.33 |
| b2925 | fbaA | fructose-bisphosphate aldolase, class II | 11.67 |
| b2610 | ffh | Signal Recognition Particle (SRP) component with 4.5S RNA (ffs) | 11.00 |
| b0684 | fldA | flavodoxin 1 | 5.00 |
| b1071 | flgM | anti-sigma factor for FliA (sigma 28) | 10.67 |
| b1937 | fliE | flagellar basal-body component | 11.67 |
| b0048 | folA | dihydrofolate reductase | 3.33 |
| b3058 | folB | dihydroneopterin aldolase and dihydroneopterin triphosphate 2'-epimerase | 7.67 |
| b2315 | folC | bifunctional folylpolyglutamate synthase/ dihydrofolate synthase | 9.00 |
| b0529 | folD | bifunctional 5,10-methylene-tetrahydrofolate dehydrogenase/ 5,10-methylene-tetrahydrofolate cyclohydrolase | 8.67 |
| b2153 | folE | GTP cyclohydrolase I | 6.67 |
| b3177 | folP | 7,8-dihydropteroate synthase | 9.33 |
| b0172 | frr | ribosome recycling factor | 4.33 |
| b2748 | ftsB | cell division protein | 4.00 |
| b3463 | ftsE | cell division ATP-binding protein | 6.67 |
| b0083 | ftsL | membrane bound cell division leucine zipper septum protein | 4.00 |
| b0093 | ftsQ | divisome assembly protein, membrane anchored protein involved in growth of wall at septum | 10.33 |
| b3462 | ftsX | putative ABC transporter permease | 7.00 |
| b0095 | ftsZ | GTP-binding tubulin-like cell division protein | 8.33 |
| b0683 | fur | ferric iron uptake regulon transcriptional repressor, autorepressor | 11.67 |
| b1779 | gapA | glyceraldehyde-3-phosphate dehydrogenase A | 8.00 |
| b0670 | glnU | tRNA-Gln | 1.33 |
| b0665 | glnV | tRNA-Gln | 2.67 |
| b0668 | glnW | tRNA-Gln | 1.33 |
| b0664 | glnX | tRNA-Gln | 4.33 |
| b3969 | gltT | tRNA-Glu | 5.00 |
| b3757 | gltU | tRNA-Glu | 5.33 |
| b4008 | gltV | tRNA-Glu | 5.00 |
| b2590 | gltW | tRNA-Glu | 3.00 |
| b2400 | gltX | glutamyl-tRNA synthetase | 10.67 |
| b2551 | glyA | serine hydroxymethyltransferase | 10.33 |
| b3560 | glyQ | glycine tRNA synthetase, alpha subunit | 7.00 |
| b3978 | glyT | tRNA-Gly | 3.67 |
| b2864 | glyU | tRNA-Gly | 10.67 |
| b4163 | glyV | tRNA-Gly | 6.67 |
| b1911 | glyW | tRNA-Gly | 4.00 |
| b4164 | glyX | tRNA-Gly | 8.00 |
| b4165 | glyY | tRNA-Gly | 8.00 |
| b3648 | gmk | guanylate kinase | 5.33 |
| b4517 | gnsA | putative phosphatidylethanolamine synthesis regulator | 8.33 |
| b1550 | gnsB | Qin prophage, multicopy suppressor of secG(Cs) and fabA6(Ts) | 4.67 |
| b3608 | gpsA | glycerol-3-phosphate dehydrogenase (NAD+) | 9.00 |
| b4142 | groS | Cpn10 chaperonin GroES, small subunit of GroESL | 6.67 |
| b2614 | grpE | heat shock protein | 4.67 |
| b0849 | grxA | glutaredoxin 1, redox coenzyme for ribonucleotide reductase (RNR1a) | 10.67 |
| b2496 | hda | ATPase regulatory factor involved in DnaA inactivation | 4.33 |
| b3805 | hemC | hydroxymethylbilane synthase | 11.00 |
| b3804 | hemD | uroporphyrinogen III synthase | 10.00 |
| b3850 | hemG | protoporphyrin oxidase, flavoprotein | 3.67 |
| b0475 | hemH | ferrochelatase | 9.00 |
| b3082 | higA | antitoxinof the HigB-HigA toxin-antitoxin system | 11.67 |
| b1508 | hipB | antitoxin of HipAB toxin-antitoxin system | 3.00 |
| b2018 | hisL | his operon leader peptide | 0.00 |
| b3797 | hisR | tRNA-His | 0.67 |
| b2514 | hisS | histidyl tRNA synthetase | 8.33 |
| b1562 | hokD | Qin prophage, small toxic polypeptide | 6.67 |
| b1099 | holB | DNA polymerase III, delta prime subunit | 9.67 |
| b4259 | holC | DNA polymerase III, chi subunit | 5.33 |
| b4372 | holD | DNA polymerase III, psi subunit | 9.67 |
| b1842 | holE | DNA polymerase III, theta subunit | 7.67 |
| b0966 | hspQ | heat shock protein involved in degradation of mutant DnaA, hemimethylated oriC DNA-binding protein | 11.33 |
| b4667 | ibsA | toxic membrane protein | 5.00 |
| b4668 | ibsB | toxic membrane protein | 3.00 |
| b4665 | ibsC | toxic membrane protein | 8.00 |
| b4664 | ibsD | toxic membrane protein | 6.00 |
| b4666 | ibsE | toxic membrane protein | 5.33 |
| b1712 | ihfA | integration host factor (IHF), DNA-binding protein, alpha subunit | 11.33 |
| b3852 | ileT | tRNA-Ile | 8.00 |
| b3277 | ileU | tRNA-Ile | 6.00 |
| b0202 | ileV | tRNA-Ile | 7.33 |
| b3069 | ileX | tRNA-Ile | 3.33 |
| b2652 | ileY | tRNA-Ile | 7.33 |
| b3766 | ilvL | ilvG operon leader peptide | 11.67 |
| b4669 | ilvX | uncharacterized protein | 8.33 |
| b0884 | infA | translation initiation factor IF-1 | 2.67 |
| b1718 | infC | translation initiation factor IF-3 | 4.00 |
| b1160 | iraM | RpoS stabilzer during Mg starvation, anti-RssB factor | 3.00 |
| b4706 | iroK | 3-hydroxypropionic acid resistance peptide | 3.67 |
| b2529 | iscU | iron-sulfur cluster assembly scaffold protein | 7.00 |
| b2524 | iscX | Fe(2+) donor and activity modulator for cysteine desulfurase | 11.67 |
| b0421 | ispA | geranyltranstransferase | 11.67 |
| b3187 | ispB | octaprenyl diphosphate synthase | 9.67 |
| b1208 | ispE | 4-diphosphocytidyl-2-C-methylerythritol kinase | 9.00 |
| b2746 | ispF | 2C-methyl-D-erythritol 2,4-cyclodiphosphate synthase | 8.33 |
| b2515 | ispG | 1-hydroxy-2-methyl-2-(E)-butenyl 4-diphosphate synthase | 9.67 |
| b0029 | ispH | 4-hydroxy-3-methylbut-2-enyl diphosphate reductase, 4Fe-4S protein | 10.00 |
| b0174 | ispU | undecaprenyl pyrophosphate synthase | 6.33 |
| b3672 | ivbL | ilvB operon leader peptide | 10.67 |
| b1215 | kdsA | 3-deoxy-D-manno-octulosonate 8-phosphate synthase | 8.33 |
| b0918 | kdsB | 3-deoxy-manno-octulosonate cytidylyltransferase | 6.67 |
| b1352 | kilR | killing protein, Rac prophage, FtsZ inhibitor protein | 4.67 |
| b4419 | ldrA | toxic polypeptide, small | 7.33 |
| b4421 | ldrB | toxic polypeptide, small | 3.00 |
| b4423 | ldrC | toxic polypeptide, small | 7.00 |
| b2568 | lepB | leader peptidase (signal peptidase I) | 9.67 |
| b4369 | leuP | tRNA-Leu | 7.33 |
| b4370 | leuQ | tRNA-Leu | 3.00 |
| b3798 | leuT | tRNA-Leu | 5.00 |
| b3174 | leuU | tRNA-Leu | 4.33 |
| b4368 | leuV | tRNA-Leu | 3.33 |
| b0672 | leuW | tRNA-Leu | 0.33 |
| b4270 | leuX | tRNA-Leu | 6.00 |
| b1909 | leuZ | tRNA-Leu | 1.33 |
| b4043 | lexA | transcriptional repressor of SOS regulon | 5.67 |
| b2828 | lgt | phosphatidylglycerol-prolipoprotein diacylglyceryl transferase | 7.00 |
| b0891 | lolA | lipoprotein chaperone | 5.67 |
| b1209 | lolB | lipoprotein localization factor | 4.00 |
| b1116 | lolC | lipoprotein-releasing system transmembrane protein | 8.00 |
| b1117 | lolD | outer membrane-specific lipoprotein transporter subunit | 7.00 |
| b1118 | lolE | lipoprotein-releasing system transmembrane protein | 10.00 |
| b1677 | lpp | murein lipoprotein | 4.00 |
| b3200 | lptA | lipopolysaccharide export ABC transporter periplasmic binding protein, Lipid A binding protein, LPS export and assembly protein | 7.67 |
| b3201 | lptB | lipopolysaccharide export ABC transporter ATPase | 7.33 |
| b0641 | lptE | LPS assembly OM complex LptDE, lipoprotein component | 4.33 |
| b4261 | lptF | lipopolysaccharide export ABC permease | 10.33 |
| b4262 | lptG | lipopolysaccharide export ABC permease | 11.00 |
| b0181 | lpxA | UDP-N-acetylglucosamine acetyltransferase | 6.33 |
| b0096 | lpxC | UDP-3-O-acyl N-acetylglucosamine deacetylase | 11.67 |
| b0179 | lpxD | UDP-3-O-(3-hydroxymyristoyl)-glucosamine N-acyltransferase | 11.00 |
| b0524 | lpxH | UDP-2,3-diacylglucosamine pyrophosphohydrolase | 5.67 |
| b0915 | lpxK | lipid A 4'kinase | 11.00 |
| b1054 | lpxL | lauryl-acyl carrier protein (ACP)-dependent acyltransferase | 7.67 |
| b0027 | lspA | prolipoprotein signal peptidase (signal peptidase II) | 4.00 |
| b0749 | lysQ | tRNA-Lys | 5.00 |
| b0743 | lysT | tRNA-Lys | 5.67 |
| b2404 | lysV | tRNA-Lys | 5.67 |
| b0745 | lysW | tRNA-Lys | 8.00 |
| b0747 | lysY | tRNA-Lys | 7.33 |
| b0748 | lysZ | tRNA-Lys | 6.67 |
| b0168 | map | methionine aminopeptidase | 8.00 |
| b1531 | marA | multiple antibiotic resistance transcriptional regulator | 10.00 |
| b1532 | marB | periplasmic mar operon regulator | 6.33 |
| b0956 | matP | Ter macrodomain organizer matS-binding protein | 11.67 |
| b0806 | mcbA | colanic acid mucoidy stimulation protein | 11.00 |
| b2942 | metK | S-adenosylmethionine synthetase | 11.33 |
| b0673 | metT | tRNA-Met | 1.67 |
| b0666 | metU | tRNA-Met | 2.33 |
| b2816 | metV | tRNA-Met | 8.00 |
| b2815 | metW | tRNA-Met | 8.00 |
| b3171 | metY | tRNA-Met | 4.33 |
| b2814 | metZ | tRNA-Met | 9.00 |
| b1826 | mgrB | regulatory peptide for PhoPQ, feedback inhibition | 5.67 |
| b1174 | minE | cell division topological specificity factor | 5.33 |
| b4705 | mntS | Mn(2)-response protein, MntR-repressed | 8.00 |
| b0087 | mraY | phospho-N-acetylmuramoyl-pentapeptide transferase | 11.00 |
| b0634 | mrdB | cell wall shape-determining protein | 8.00 |
| b3251 | mreB | cell wall structural complex MreBCD, actin-like component MreB | 7.33 |
| b3249 | mreD | cell wall structural complex MreBCD transmembrane component MreD | 3.00 |
| b1778 | msrB | methionine sulfoxide reductase B | 11.33 |
| b0923 | mukE | chromosome condensin MukBEF, MukE localization factor | 6.00 |
| b0922 | mukF | chromosome condensin MukBEF, kleisin-like subunit, binds calcium | 11.67 |
| b3189 | murA | UDP-N-acetylglucosamine 1-carboxyvinyltransferase | 7.33 |
| b0086 | murF | UDP-N-acetylmuramoyl-tripeptide:D-alanyl-D-alanine ligase | 11.00 |
| b0090 | murG | N-acetylglucosaminyl transferase | 6.67 |
| b3967 | murI | glutamate racemase | 6.33 |
| b0639 | nadD | nicotinic acid mononucleotide adenylyltransferase, NAD(P)-dependent | 9.33 |
| b1740 | nadE | NAD synthetase, NH3/glutamine-dependent | 7.67 |
| b2615 | nadK | NAD kinase | 7.33 |
| b2207 | napD | assembly protein for periplasmic nitrate reductase | 11.33 |
| b2673 | nrdH | hydrogen donor for NrdEF electron transport system, glutaredoxin-like protein | 11.33 |
| b0416 | nusB | transcription antitermination protein | 8.33 |
| b3982 | nusG | transcription termination factor | 7.00 |
| b4162 | orn | oligoribonuclease | 6.33 |
| b1638 | pdxH | pyridoxine 5'-phosphate oxidase | 6.00 |
| b1912 | pgsA | phosphatidylglycerophosphate synthetase | 5.33 |
| b2598 | pheL | pheA gene leader peptide | 2.33 |
| b1715 | pheM | phenylalanyl-tRNA synthetase operon leader peptide | 0.00 |
| b1714 | pheS | phenylalanine tRNA synthetase, alpha subunit | 10.00 |
| b4134 | pheU | tRNA-Phe | 4.00 |
| b2967 | pheV | tRNA-Phe | 6.67 |
| b3018 | plsC | 1-acyl-sn-glycerol-3-phosphate acyltransferase | 8.67 |
| b4703 | pmrR | putative membrane-bound BasS regulator | 9.67 |
| b4226 | ppa | inorganic pyrophosphatase | 8.33 |
| b0525 | ppiB | peptidyl-prolyl cis-trans isomerase B (rotamase B) | 11.00 |
| b1211 | prfA | peptide chain release factor RF-1 | 8.00 |
| b4201 | priB | primosomal protein N | 3.00 |
| b1212 | prmC | RF-1 and RF-2 N5-glutamine methyltransferase | 6.67 |
| b3545 | proK | tRNA-Pro | 3.33 |
| b2189 | proL | tRNA-Pro | 2.00 |
| b3799 | proM | tRNA-Pro | 1.67 |
| b1207 | prs | phosphoribosylpyrophosphate synthase | 6.33 |
| b1305 | pspB | psp operon transcription co-activator | 7.67 |
| b1306 | pspC | psp operon transcription co-activator | 10.67 |
| b1307 | pspD | peripheral inner membrane phage-shock protein | 4.67 |
| b2585 | pssA | phosphatidylserine synthase, CDP-diacylglycerol-serine O-phosphatidyltransferase | 8.33 |
| b1204 | pth | peptidyl-tRNA hydrolase | 7.33 |
| b2415 | ptsH | phosphohistidinoprotein-hexose phosphotransferase component of PTS system (Hpr) | 10.33 |
| b0171 | pyrH | uridylate kinase | 9.00 |
| b1351 | racC | Rac prophage, uncharacterized protein | 8.00 |
| b1356 | racR | Rac prophage, putative DNA-binding transcriptional regulator | 5.33 |
| b1348 | ralR | Rac prophage, restriction alleviation protein | 5.67 |
| b2618 | ratB | UPF0125 family protein | 11.00 |
| b3167 | rbfA | 30s ribosome binding factor | 11.33 |
| b1347 | rcbA | DUF1187 family protein, Rac prophage, putative double-strand break reduction protein | 0.67 |
| b1564 | relB | antitoxin of the RelE-RelB toxin-antitoxin syste, transcriptional repressor | 7.67 |
| b1563 | relE | Qin prophage, toxin of the RelE-RelB toxin-antitoxin system | 9.00 |
| b1277 | ribA | GTP cyclohydrolase II | 5.33 |
| b1662 | ribC | riboflavin synthase, alpha subunit | 7.00 |
| b0415 | ribE | riboflavin synthase beta chain | 3.00 |
| b2608 | rimM | ribosome maturation factor | 6.00 |
| b2594 | rluD | 23S rRNA pseudouridine(1911,1915,1917) synthase | 9.67 |
| b0953 | rmf | ribosome modulation factor | 11.67 |
| b3704 | rnpA | protein C5 component of RNase P | 6.67 |
| b3984 | rplA | 50S ribosomal subunit protein L1 | 11.00 |
| b3317 | rplB | 50S ribosomal subunit protein L2 | 6.33 |
| b3320 | rplC | 50S ribosomal subunit protein L3 | 5.00 |
| b3319 | rplD | 50S ribosomal subunit protein L4 | 7.33 |
| b3308 | rplE | 50S ribosomal subunit protein L5 | 5.33 |
| b3305 | rplF | 50S ribosomal subunit protein L6 | 4.67 |
| b3985 | rplJ | 50S ribosomal subunit protein L10 | 4.00 |
| b3983 | rplK | 50S ribosomal subunit protein L11 | 3.00 |
| b3986 | rplL | 50S ribosomal subunit protein L7/L12 | 4.00 |
| b3231 | rplM | 50S ribosomal subunit protein L13 | 4.33 |
| b3310 | rplN | 50S ribosomal subunit protein L14 | 2.33 |
| b3301 | rplO | 50S ribosomal subunit protein L15 | 3.67 |
| b3313 | rplP | 50S ribosomal subunit protein L16 | 1.67 |
| b3294 | rplQ | 50S ribosomal subunit protein L17 | 4.67 |
| b3304 | rplR | 50S ribosomal subunit protein L18 | 2.33 |
| b2606 | rplS | 50S ribosomal subunit protein L19 | 3.33 |
| b1716 | rplT | 50S ribosomal subunit protein L20 | 2.67 |
| b3186 | rplU | 50S ribosomal subunit protein L21 | 3.00 |
| b3315 | rplV | 50S ribosomal subunit protein L22 | 3.67 |
| b3318 | rplW | 50S ribosomal subunit protein L23 | 2.33 |
| b3309 | rplX | 50S ribosomal subunit protein L24 | 1.67 |
| b2185 | rplY | 50S ribosomal subunit protein L25 | 2.67 |
| b3185 | rpmA | 50S ribosomal subunit protein L27 | 4.67 |
| b3637 | rpmB | 50S ribosomal subunit protein L28 | 2.33 |
| b3312 | rpmC | 50S ribosomal subunit protein L29 | 2.33 |
| b3302 | rpmD | 50S ribosomal subunit protein L30 | 2.33 |
| b3936 | rpmE | 50S ribosomal subunit protein L31 | 11.33 |
| b1089 | rpmF | 50S ribosomal subunit protein L32 | 5.33 |
| b3636 | rpmG | 50S ribosomal subunit protein L33 | 6.67 |
| b3703 | rpmH | 50S ribosomal subunit protein L34 | 0.67 |
| b1717 | rpmI | 50S ribosomal subunit protein L35 | 1.67 |
| b3299 | rpmJ | 50S ribosomal subunit protein L36 | 0.33 |
| b3295 | rpoA | RNA polymerase, alpha subunit | 7.33 |
| b2573 | rpoE | RNA polymerase sigma E factor | 5.67 |
| b3461 | rpoH | RNA polymerase, sigma 32 (sigma H) factor | 7.00 |
| b0169 | rpsB | 30S ribosomal subunit protein S2 | 9.00 |
| b3314 | rpsC | 30S ribosomal subunit protein S3 | 10.00 |
| b3296 | rpsD | 30S ribosomal subunit protein S4 | 6.67 |
| b3303 | rpsE | 30S ribosomal subunit protein S5 | 6.00 |
| b4200 | rpsF | 30S ribosomal subunit protein S6 | 4.00 |
| b3341 | rpsG | 30S ribosomal subunit protein S7 | 5.00 |
| b3306 | rpsH | 30S ribosomal subunit protein S8 | 3.33 |
| b3230 | rpsI | 30S ribosomal subunit protein S9 | 3.67 |
| b3321 | rpsJ | 30S ribosomal subunit protein S10 | 3.67 |
| b3297 | rpsK | 30S ribosomal subunit protein S11 | 2.67 |
| b3342 | rpsL | 30S ribosomal subunit protein S12 | 3.33 |
| b3298 | rpsM | 30S ribosomal subunit protein S13 | 3.00 |
| b3307 | rpsN | 30S ribosomal subunit protein S14 | 1.67 |
| b3165 | rpsO | 30S ribosomal subunit protein S15 | 2.67 |
| b2609 | rpsP | 30S ribosomal subunit protein S16 | 2.67 |
| b3311 | rpsQ | 30S ribosomal subunit protein S17 | 2.67 |
| b4202 | rpsR | 30S ribosomal subunit protein S18 | 7.33 |
| b3316 | rpsS | 30S ribosomal subunit protein S19 | 2.67 |
| b0023 | rpsT | 30S ribosomal subunit protein S20 | 2.67 |
| b3065 | rpsU | 30S ribosomal subunit protein S21 | 2.00 |
| b3272 | rrfF | 5S ribosomal RNA of rrnD operon | 10.67 |
| b0176 | rseP | inner membrane zinc RIP metalloprotease, RpoE activator, by degrading RseA, cleaved signal peptide endoprotease | 10.67 |
| b3609 | secB | protein export chaperone | 7.67 |
| b3981 | secE | preprotein translocase membrane subunit | 2.33 |
| b0409 | secF | SecYEG protein translocase auxillary subunit | 9.33 |
| b0097 | secM | regulator of secA translation | 9.67 |
| b0687 | seqA | negative modulator of initiation of replication | 4.33 |
| b0971 | serT | tRNA-Ser | 2.00 |
| b1975 | serU | tRNA-Ser | 3.00 |
| b2695 | serV | tRNA-Ser | 1.67 |
| b0883 | serW | tRNA-Ser | 3.67 |
| b1032 | serX | tRNA-Ser | 2.67 |
| b1480 | sra | stationary-phase-induced ribosome-associated protein | 5.00 |
| b4059 | ssb | single-stranded DNA-binding protein | 2.00 |
| b2533 | suhB | inositol monophosphatase | 5.33 |
| b2559 | tadA | tRNA-specific adenosine deaminase | 6.67 |
| b0417 | thiL | thiamine monophosphate kinase | 10.67 |
| b0001 | thrL | thr operon leader peptide | 6.67 |
| b3979 | thrT | tRNA-Thr | 3.67 |
| b3976 | thrU | tRNA-Thr | 1.33 |
| b3273 | thrV | tRNA-Thr | 3.33 |
| b0244 | thrW | tRNA-Thr | 2.67 |
| b2827 | thyA | thymidylate synthetase | 10.67 |
| b4618 | tisB | toxic membrane persister formation peptide, LexA-regulated | 11.67 |
| b1098 | tmk | thymidylate kinase | 8.67 |
| b3707 | tnaC | tryptophanase leader peptide | 10.00 |
| b1229 | tpr | protamine-like protein | 5.00 |
| b2607 | trmD | tRNA m(1)G37 methyltransferase, SAM-dependent | 11.00 |
| b1265 | trpL | trp operon leader peptide | 1.33 |
| b3384 | trpS | tryptophanyl-tRNA synthetase | 9.33 |
| b3761 | trpT | tRNA-Trp | 1.67 |
| b2582 | trxC | thioredoxin 2 | 11.33 |
| b1807 | tsaB | tRNA(ANN) t(6)A37 threonylcarbamoyladenosine modification protein, binding partner and protease for TsaD | 3.33 |
| b3282 | tsaC | tRNA(ANN) t(6)A37 threonylcarbamoyladenosine modification protein, threonine-dependent ADP-forming ATPase | 8.00 |
| b3064 | tsaD | tRNA(ANN) t(6)A37 threonylcarbamoyladenosine modification protein, glycation binding protein | 11.33 |
| b4168 | tsaE | tRNA(ANN) t(6)A37 threonylcarbamoyladenosine modification protein, ADP binding protein | 7.33 |
| b0170 | tsf | translation elongation factor EF-Ts | 6.33 |
| b3343 | tusB | mnm(5)-s(2)U34-tRNA synthesis 2-thiolation protein | 11.33 |
| b0969 | tusE | mnm(5)-s(2)U34-tRNA 2-thiolation sulfurtransferase | 10.33 |
| b1231 | tyrT | tRNA-Tyr | 2.67 |
| b3977 | tyrU | tRNA-Tyr | 2.67 |
| b1230 | tyrV | tRNA-Tyr | 2.67 |
| b3833 | ubiE | bifunctional 2-octaprenyl-6-methoxy-1,4-benzoquinone methylase/ S-adenosylmethionine:2-DMK methyltransferase | 10.33 |
| b2232 | ubiG | bifunctional 3-demethylubiquinone-9 3-methyltransferase/ 2-octaprenyl-6-hydroxy phenol methylase | 7.67 |
| b2311 | ubiX | 3-octaprenyl-4-hydroxybenzoate carboxy-lyase | 5.33 |
| b4637 | uof | ryhB-regulated fur leader peptide | 5.00 |
| b0744 | valT | tRNA-Val | 5.67 |
| b2401 | valU | tRNA-Val | 4.67 |
| b1665 | valV | tRNA-Val | 1.67 |
| b1666 | valW | tRNA-Val | 2.67 |
| b2402 | valX | tRNA-Val | 5.67 |
| b2403 | valY | tRNA-Val | 4.00 |
| b0746 | valZ | tRNA-Val | 5.33 |
| b0422 | xseB | exonuclease VII small subunit | 7.67 |
| b0024 | yaaY | uncharacterized protein | 8.33 |
| b0101 | yacG | DNA gyrase inhibitor | 9.33 |
| b4406 | yaeP | UPF0253 family protein | 11.00 |
| b0232 | yafN | antitoxin of the YafO-YafN toxin-antitoxin system | 10.33 |
| b0407 | yajC | SecYEG protein translocase auxillary subunit | 8.67 |
| b0498 | ybbC | putative immunity protein | 8.67 |
| b0558 | ybcV | DLP12 prophage, uncharacterized protein | 9.33 |
| b4512 | ybdD | DUF466 family protein | 9.33 |
| b0631 | ybeD | UPF0250 family protein | 7.00 |
| b0659 | ybeY | ssRNA-specific endoribonuclease, 16S rRNA 3' end maturation and quality control co-endoribonuclease working with RNase R, rRNA transcription antitermination factor | 6.33 |
| b0702 | ybfB | putative membrane protein | 11.67 |
| b0704 | ybfC | putative secreted protein | 8.00 |
| b4515 | ybgT | cytochrome d (bd-I) ubiquinol oxidase subunit X | 3.00 |
| b0762 | ybhT | AcrAB-TolC efflux pump accessory protein, membrane-associated | 7.33 |
| b0802 | ybiJ | DUF1471 family putative periplasmic protein | 8.33 |
| b0917 | ycaR | peroxide and acid resistance protein, UPF0434 family | 2.67 |
| b4592 | yccB | putative cytochrome bd-II oxidase subunit | 9.33 |
| b1003 | yccJ | uncharacterized protein | 11.00 |
| b1058 | yceO | uncharacterized protein | 6.00 |
| b1085 | yceQ | uncharacterized protein | 7.00 |
| b1257 | yciE | putative rubrerythrin/ferritin-like metal-binding protein | 6.00 |
| b1259 | yciG | KGG family protein | 1.67 |
| b1280 | yciM | LPS regulatory protein, putative modulator of LpxC proteolysis | 10.33 |
| b1279 | yciS | DUF1049 family inner membrane protein, function unknown | 6.33 |
| b1248 | yciU | UPF0263 family protein | 7.67 |
| b4523 | yciX | uncharacterized protein | 10.33 |
| b4595 | yciY | uncharacterized protein | 10.67 |
| b4596 | yciZ | uncharacterized protein | 10.33 |
| b4526 | ydaE | conserved protein, Rac prophage | 0.33 |
| b1355 | ydaG | Rac prophage, uncharacterized protein | 7.33 |
| b1419 | ydcA | putative periplasmic protein | 11.67 |
| b1457 | ydcD | putative immunity protein for RhsE | 6.33 |
| b1445 | ydcX | DUF2566 family protein | 4.33 |
| b1446 | ydcY | DUF2526 family protein | 6.00 |
| b1477 | yddM | putative DNA-binding transcriptional regulator | 6.00 |
| b1536 | ydeI | hydrogen peroxide resistance OB fold protein, putative periplasmic protein | 8.67 |
| b1571 | ydfA | Qin prophage, uncharacterized protein | 4.00 |
| b1572 | ydfB | Qin prophage, uncharacterized protein | 4.67 |
| b1544 | ydfK | cold shock protein, function unknown, Qin prophage | 7.33 |
| b1549 | ydfO | Qin prophage, uncharacterized protein | 6.67 |
| b1555 | ydfR | Qin prophage, uncharacterized protein | 10.67 |
| b1541 | ydfZ | selenoprotein, function unknown | 8.00 |
| b4601 | ydgU | stationary phase-induced protein | 4.33 |
| b1648 | ydhL | DUF1289 family protein | 10.00 |
| b1667 | ydhR | putative monooxygenase | 5.67 |
| b1705 | ydiE | hemin uptake protein HemP homolog | 5.00 |
| b1685 | ydiH | uncharacterized protein | 11.33 |
| b1724 | ydiZ | uncharacterized protein | 11.00 |
| b1848 | yebG | DNA damage-inducible protein regulated by LexA | 8.33 |
| b1836 | yebV | uncharacterized protein | 11.67 |
| b1837 | yebW | uncharacterized protein | 9.33 |
| b4537 | yecJ | DUF2766 family protein | 6.33 |
| b2012 | yeeD | putative TusA family sulfurtransferase | 11.00 |
| b2007 | yeeX | UPF0265 family protein | 2.67 |
| b2017 | yefM | antitoxin of the YoeB-YefM toxin-antitoxin system | 7.00 |
| b2110 | yehC | putative periplasmic pilin chaperone | 2.67 |
| b2111 | yehD | putative fimbrial-like adhesin protein | 7.33 |
| b4502 | yeiW | UPF0153 cysteine cluster protein | 8.67 |
| b2181 | yejG | uncharacterized protein | 8.00 |
| b2187 | yejL | UPF0352 family protein | 5.67 |
| b2273 | yfbN | uncharacterized protein | 9.00 |
| b2444 | yffM | CPZ-55 prophage, uncharacterized protein | 10.33 |
| b2510 | yfgJ | DUF1407 family protein | 11.00 |
| b2579 | yfiD | autonomous glycyl radical cofactor | 7.67 |
| b2851 | ygeG | SycD-like chaperone family TPR-repeat-containing protein | 5.33 |
| b2853 | ygeI | uncharacterized protein | 1.33 |
| b3107 | yhaL | uncharacterized protein | 8.00 |
| b3865 | yihA | cell division GTP-binding protein | 8.00 |
| b4011 | yjaA | stress-induced protein | 11.33 |
| b4621 | yjbS | uncharacterized protein | 4.00 |
| b4128 | yjdK | antitoxin of GhoTS toxin-antitoxin pair, endonuclease for ghoT mRNA | 9.00 |
| b4670 | yjeV | uncharacterized protein | 2.33 |
| b4586 | ykfM | lethality reduction protein, putative inner membrane protein | 5.00 |
| b0310 | ykgH | putative inner membrane protein | 11.00 |
| b0303 | ykgI | reactive chlorine species (RCS) stress resistance periplasmic protein | 5.33 |
| b4506 | ykgO | RpmJ-like protein | 10.33 |
| b4671 | ykgR | uncharacterized protein | 7.00 |
| b1137 | ymfD | e14 prophage, putative SAM-dependent methyltransferase | 8.00 |
| b1138 | ymfE | e14 prophage, putative inner membrane protein | 7.00 |
| b1165 | ymgA | RcsB connector protein for regulation of biofilm | 4.67 |
| b1167 | ymgC | Blue light, low temperature and stress induced protein | 6.00 |
| b4593 | ymgI | uncharacterized protein | 6.00 |
| b4522 | ymiA | uncharacterized protein | 9.00 |
| b4672 | ymiB | uncharacterized protein | 1.00 |
| b1375 | ynaE | cold shock protein, Rac prophage | 6.33 |
| b4674 | ynbG | uncharacterized protein | 0.67 |
| b1455 | yncH | IPR020099 family protein | 1.00 |
| b1436 | yncJ | uncharacterized protein | 8.00 |
| b4598 | yncL | stress-induced small inner membrane enterobacterial protein | 4.33 |
| b4599 | yneM | inner membrane-associated protein | 6.67 |
| b1500 | yneN | two-component system connector membrane protein, EvgSA to PhoQP | 2.00 |
| b1551 | ynfN | Qin prophage, cold shock-induced protein | 2.00 |
| b4602 | ynhF | stress response membrane | 4.33 |
| b1793 | yoaF | DUF333 family outer membrane lipoprotein | 10.00 |
| b1788 | yoaI | uncharacterized protein | 5.00 |
| b4675 | yoaJ | inner membrane-associated protein | 8.00 |
| b4676 | yoaK | inner membrane-associated protein | 5.33 |
| b4677 | yobI | uncharacterized protein | 4.00 |
| b4678 | yoeI | uncharacterized protein | 4.67 |
| b4542 | yohO | putative membrane protein | 5.67 |
| b4679 | yohP | uncharacterized protein | 5.00 |
| b4680 | ypdK | inner membrane protein | 1.33 |
| b4606 | ypfM | stress-induced small enterobacterial protein | 6.33 |
| b4547 | ypfN | putative membrane protein, UPF0370 family | 10.00 |
| b4682 | yqcG | membrane stress resistance protein | 2.00 |
| b2848 | yqeJ | uncharacterized protein | 5.67 |
| b2849 | yqeK | uncharacterized protein | 3.67 |
| b4683 | yqeL | uncharacterized protein | 1.33 |
| b2949 | yqgF | putative Holliday junction resolvase | 10.00 |
| b4685 | yrbN | uncharacterized protein | 5.67 |
| b2412 | zipA | FtsZ stabilizer | 9.67 |
| b4714 |  | Pseudo gene | 0.67 |

**Supplementary Table 2.** List of genes with strong to moderate log-fold decreases in sequencing reads in Δ*recG*, representing potential *recG* genetic interactions

| **Gene ID** | **Gene Name** | **Annotation** | **Log_2_-fold change in sequencing reads** |
| --- | --- | --- | --- |
| b3387 | *dam* | DNA adenine methyltransferase | -7.55 |
| b3813 | *uvrD* | DNA-dependent ATPase I and helicase II | -6.69 |
| b0214 | *rnhA* | ribonuclease HI, degrades RNA of DNA-RNA hybrids | -5.51 |
| b4389 | *radA* | DNA repair protein | -4.66 |
| b3778 | *rep* | DNA helicase and single-stranded DNA-dependent ATPase | -4.13 |
| b3066 | *dnaG* | DNA primase | -4.08 |
| b4484 | *cpxP* | inhibitor of the cpx response, periplasmic adaptor protein | -3.74 |
| b3261 | *fis* | global DNA-binding transcriptional dual regulator | -3.55 |
| b4686 | *yshB* | uncharacterized protein | -2.94 |
| b3229 | *sspA* | stringent starvation protein A, phage P1 late gene activator, RNAP-associated acid-resistance protein, inactive glutathione S-transferase homolog | -2.85 |
| b3738 | *atpB* | F0 sector of membrane-bound ATP synthase, subunit a | -2.70 |
| b3344 | *tusC* | mnm(5)-s(2)U34-tRNA synthesis 2-thiolation protein | -2.69 |
| b0627 | *tatE* | TatABCE protein translocation system subunit | -2.68 |
| b3919 | *tpiA* | triosephosphate isomerase | -2.58 |
| b3944 | *yijF* | DUF1287 family protein | -2.53 |
| b3935 | *priA* | Primosome factor n' (replication factor Y) | -2.49 |
| b3855 | *rrfA* | 5S ribosomal RNA of rrnA operon | -2.49 |
| b0890 | *ftsK* | DNA translocase at septal ring sorting daughter chromsomes | -2.49 |
| b0439 | *lon* | DNA-binding ATP-dependent protease La | -2.46 |
| b3863 | *polA* | 5' to 3' DNA polymerase and 3' to 5'/5' to 3' exonuclease | -2.40 |
| b0657 | *lnt* | apolipoprotein N-acyltransferase | -2.39 |
| b3250 | *mreC* | cell wall structural complex MreBCD transmembrane component MreC | -2.36 |
| b2525 | *fdx* | [2Fe-2S] ferredoxin | -2.35 |
| b4482 | *yigE* | DUF2233 family protein | -2.32 |
| b3382 | *yhfY* | PRD domain protein | -2.30 |
| b2328 | *mepA* | murein DD-endopeptidase | -2.28 |
| b2954 | *rdgB* | dITP/XTP pyrophosphatase | -2.22 |
| b4172 | *hfq* | global sRNA chaperone, HF-I, host factor for RNA phage Q beta replication | -2.21 |
| b2184 | *radD* | putative ATP-dependent DNA or RNA helicase | -2.16 |
| b4092 | *phnP* | 5-phospho-alpha-D-ribosyl 1,2-cyclic phosphate phosphodiesterase | -2.16 |
| b3497 | *yhiQ* | 16S rRNA m(2)G1516 methyltransferase, SAM-dependent | -2.14 |
| b3729 | *glmS* | L-glutamine:D-fructose-6-phosphate aminotransferase | -2.12 |
| b3769 | *ilvM* | acetolactate synthase 2 small subunit | -2.09 |
| b3450 | *ugpC* | sn-glycerol-3-phosphate ABC transporter ATPase | -2.06 |
| b4147 | *efp* | polyproline-specific translation elongation factor EF-P | -2.02 |
| b3472 | *dcrB* | putative lipoprotein | -2.01 |
| b3701 | *dnaN* | DNA polymerase III, beta subunit | -2.01 |
| b3950 | *frwB* | putative enzyme IIB component of PTS | -1.96 |
| b3131 | *agaR* | transcriptional repressor of the aga regulon | -1.92 |
| b4479 | *dgoR* | D-galactonate catabolism operon transcriptional repressor | -1.88 |
| b3811 | *xerC* | site-specific tyrosine recombinase | -1.88 |
| b3859 | *rdoA* | Cpx stress response Thr/Ser protein kinase, MazF antagonist protein | -1.88 |
| b4453 | *ldrD* | toxic polypeptide, small | -1.87 |
| b4034 | *malE* | maltose transporter subunit | -1.85 |
| b3162 | *deaD* | ATP-dependent RNA helicase | -1.85 |
| b2897 | *ygfY* | flavinator of succinate dehydrogenase, antitoxin of CptAB toxin-antitoxin pair | -1.84 |
| b3925 | *glpX* | fructose 1,6-bisphosphatase II | -1.83 |
| b3781 | *trxA* | thioredoxin 1 | -1.82 |
| b3759 | *rrfC* | 5S ribosomal RNA of rrnC operon | -1.82 |
| b3260 | *dusB* | tRNA-dihydrouridine synthase B | -1.81 |
| b0998 | *torD* | TorA-maturation chaperone | -1.81 |
| b3288 | *fmt* | 10-formyltetrahydrofolate:L-methionyl-tRNA(fMet) N-formyltransferase | -1.81 |
| b3528 | *dctA* | C4-dicarboxylic acid, orotate and citrate transporter | -1.81 |
| b3206 | *npr* | phosphohistidinoprotein-hexose phosphotransferase component of N-regulated PTS system (Npr) | -1.80 |
| b2434 | *ypeA* | GNAT family putative N-acetyltransferase | -1.79 |
| b4073 | *nrfD* | formate-dependent nitrite reductase, membrane subunit | -1.78 |
| b2631 | *rnlB* | CP4-57 prophage, uncharacterized protein | -1.78 |
| b3758 | *rrlC* | 23S ribosomal RNA of rrnC operon | -1.77 |
| b2236 | *yfaE* | ferredoxin involved with ribonucleotide reductase diferric-tyrosyl radical (Y*) cofactor maintenance | -1.77 |
| b3741 | *mnmG* | 5-methylaminomethyl-2-thiouridine modification at tRNA U34 | -1.76 |
| b3882 | *yihU* | 3-sulpholactaldehyde (SLA) reductase, NADH-dependent,  gamma-hydroxybutyrate dehydrogenase, NADH-dependent | -1.76 |
| b3454 | *livF* | branched-chain amino acid ABC transporter ATPase | -1.74 |
| b3259 | *prmA* | methyltransferase for 50S ribosomal subunit protein L11 | -1.74 |
| b2890 | *lysS* | lysine tRNA synthetase, constitutive | -1.74 |
| b3425 | *glpE* | thiosulfate:cyanide sulfurtransferase (rhodanese) | -1.73 |
| b2508 | *guaB* | IMP dehydrogenase | -1.73 |
| b0205 | *rrfH* | 5S ribosomal RNA of rrnH operon | -1.72 |
| b3725 | *pstB* | phosphate ABC transporter ATPase | -1.71 |
| b4009 | *rrlE* | 23S ribosomal RNA of rrnE operon | -1.71 |
| b4102 | *phnF* | putative DNA-binding transcriptional regulator of phosphonate uptake and biodegradation | -1.71 |
| b3970 | *rrlB* | 23S ribosomal RNA of rrnB operon | -1.71 |
| b0118 | *acnB* | aconitate hydratase 2, aconitase B, 2-methyl-cis-aconitate hydratase | -1.70 |
| b3412 | *bioH* | pimeloyl-ACP methyl ester carboxylesterase | -1.69 |
| b2589 | *rrlG* | 23S ribosomal RNA of rrnG operon | -1.69 |
| b3180 | *yhbY* | RNA binding protein associated with pre-50S ribosomal subunits | -1.69 |
| b2485 | *hyfE* | hydrogenase 4, membrane subunit | -1.68 |
| b3980 | *tufB* | translation elongation factor EF-Tu 2 | -1.68 |
| b3854 | *rrlA* | 23S ribosomal RNA of rrnA operon | -1.67 |
| b3275 | *rrlD* | 23S ribosomal RNA of rrnD operon | -1.67 |
| b3583 | *yiaS* | L-ribulose-5-phosphate 4-epimerase | -1.66 |
| b3693 | *dgoK* | 2-oxo-3-deoxygalactonate kinase | -1.66 |
| b3893 | *fdoH* | formate dehydrogenase-O, Fe-S subunit | -1.65 |
| b3995 | *rsd* | stationary phase protein, binds sigma 70 RNA polymerase subunit | -1.64 |
| b4058 | *uvrA* | ATPase and DNA damage recognition protein of nucleotide excision repair complex UvrABC | -1.63 |

Cells marked in yellow represent known *recG* genetic interactions.


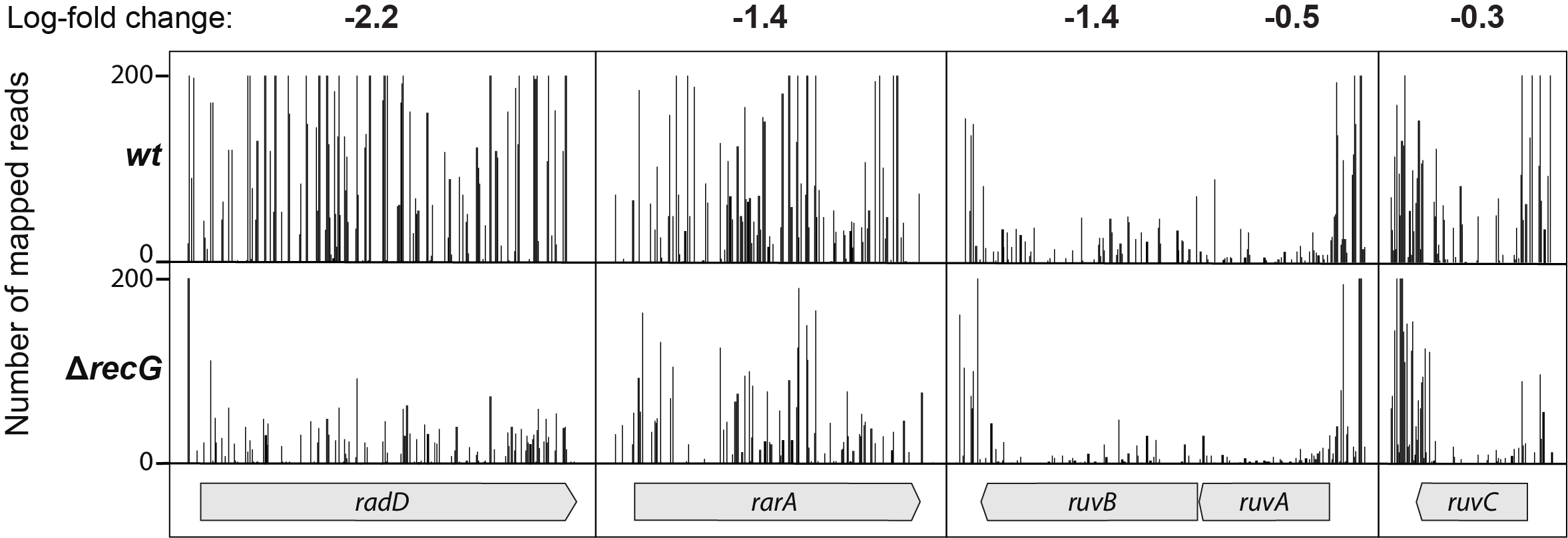


**Supplementary Figure 1**. Transposon insertion profiles for *radD, rarA,* and *ruvABC*.


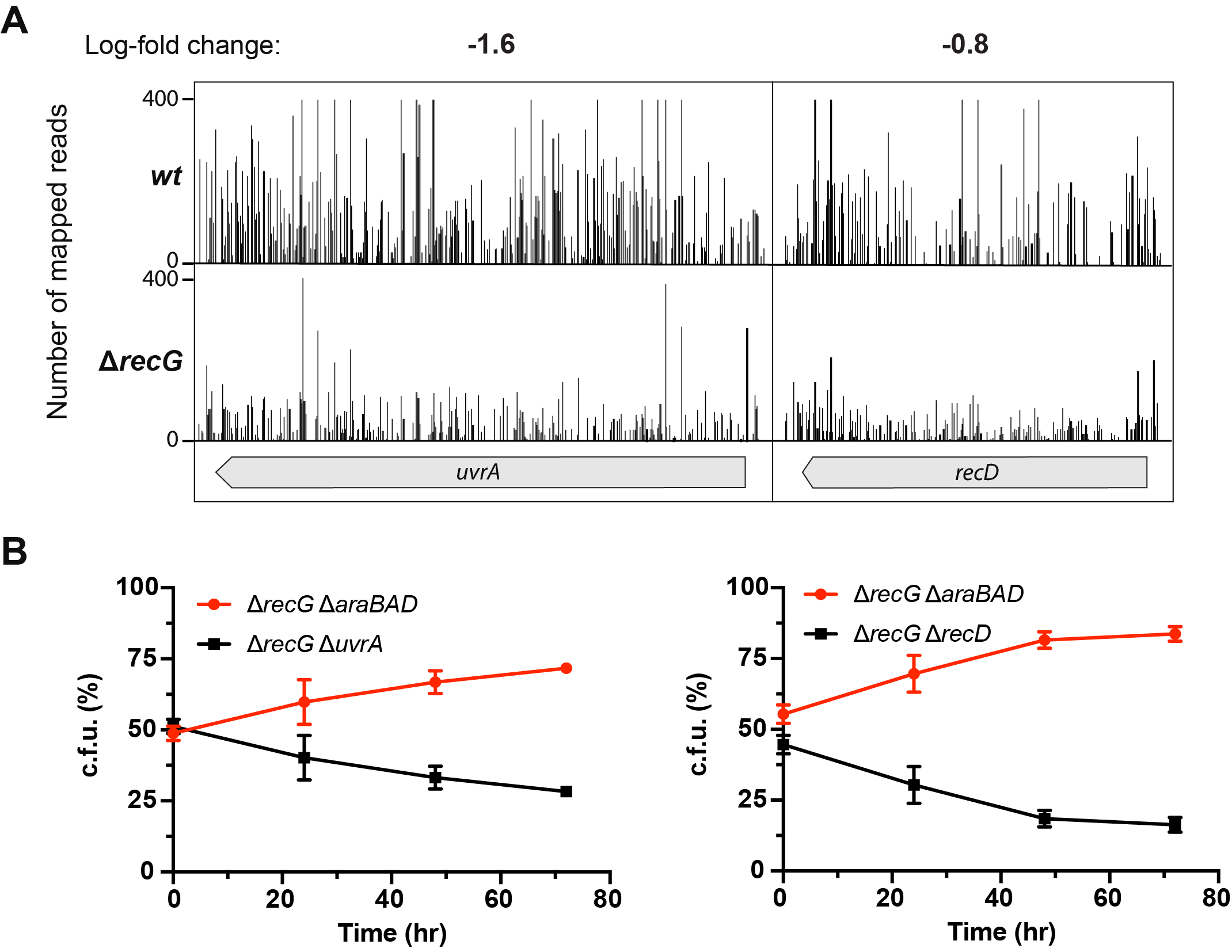


**Supplementary Figure 2.** Modest genetic interactions between *recG* and *uvrA*, *recD*. (A) Insertion profiles and log_2_-fold changes in sequencing reads for the *uvrA* and *recD* genes in the wild type and Δ*recG* libraries. (B) Percent colony forming units (c.f.u.) of Δ*recG* Δ*araBAD* versus Δ*recG* Δ*uvrA* or Δ*recG* Δ*recD* strains from competitive fitness assays. Assays were carried out in biological triplicate with the mean values plotted and error bars presenting one standard deviation away from the mean.
